# Soil microbial load modulation improves plant–microbe interactions and bioinoculant efficacy in pathogen-stressed soils

**DOI:** 10.3389/fpls.2025.1712997

**Published:** 2025-12-04

**Authors:** Yohannes Ebabuye Andargie, GyuDae Lee, Min-Ji Kim, Eskindir Getachew Fentie, Minsoo Jeong, Setu Bazie Tagele, Kyeongmo Lim, Ugur Azizoglu, Jae-Ho Shin

**Affiliations:** 1 Next-Generation Sequencing (NGS) Core Facility, Kyungpook National University, Daegu, Republic of Korea; 2 Department of Plant Sciences, Bahir Dar University, Bahir Dar, Ethiopia; 3 Department of Applied Biosciences, Kyungpook National University, Daegu, Republic of Korea; 4 Department of Food Science and Nutrition, Pukyong National University, Busan, Republic of Korea; 5 Department of Microbiology and Plant Pathology, University of California, Riverside, CA, United States; 6 Department of Crop and Animal Production, Safye Cikrikcioglu Vocational College, Kayseri University, Kayseri, Türkiye; 7 Genome and Stem Cell Research Center, Erciyes University, Kayseri, Türkiye; 8 Department of Integrative Biology, Kyungpook National University, Daegu, Republic of Korea

**Keywords:** bioinoculants, cucumber, dysbiosis, microbiome, rhizosphere, transcriptomics

## Abstract

Plants establish a close association with a community of microbes naturally living in the soil, known as resident soil microbiome, which typically maintains a dynamic equilibrium that confers resilience against biotic and abiotic perturbations. However, this microbiome can also reduce the success of adding new helpful microbes (bioinoculants) by reducing their functional integration with the host plant. Although bioinoculants often perform well under controlled conditions, their efficacy in pathogenic soils is frequently compromised even after repeated applications. While several factors influencing inoculation success have been examined, the impact of soil microbial load, its dynamics, and associated transcriptomic consequences remain largely overlooked. To address this gap, we induced dysbiosis in the resident soil microbiome using moist heat treatment (MHT) thereby generating a gradient in microbial load. We then assessed the phenotypic and transcriptomic responses of *Cucumis sativus* L., for bioinoculants alongside relative and quantitative rhizosphere microbiome profiling. MHT reduced resident soil bacterial abundance by 96.4% ± 0.9%, with 78% recovery observed after planting. This recolonization promoted plant growth and overall health by restructuring the rhizosphere microbiome and activating plant-microbe interaction pathways such as sugar metabolism, nitrogen metabolism, and aromatic compound degradation. In contrast, moist heat untreated (native) rhizosphere, with a microbial load threefold higher, resisted restructuring, favoring metabolic pathways that preserve microbial stability, such as cell wall and signal molecule biosynthesis, at the expense of plant health. Transcriptomic analyses revealed that, in moist heat treated (dysbiotic) soil conditions, bioagent inoculation triggered induced systemic resistance in cucumber, characterized by downregulation of PAL and POX gene families together with SAMDC, and upregulation of auxin-regulatory and calcium uniporter genes. This response reflected a reallocation of metabolic energy from defense to growth, while maintaining active signaling for beneficial colonization and pathogen perception via modulation of calcium influx. Our findings highlight microbial load modulation as a key strategy to facilitate rhizosphere remodeling, enhance bioinoculant efficacy, and promote plant transcriptomic responses.

## Introduction

The resident soil microbiome comprises a diverse community of soil-dwelling microorganisms that maintain a dynamic equilibrium through microbial interactions, supporting ecosystem function and stability (Osburn et al., 2023). Plants exert strong selective pressure on the resident soil microbiota through root exudate deposition, thereby assembling a rhizosphere microbiome to sustain their growth and health (Zhalnina et al., 2018). While various factors influence the assembly of the pathogen suppressive rhizosphere microbiome (Andargie et al., 2023), plants generally promote the recruitment of beneficial microbes while suppressing pathogen colonization (Teixeira et al., 2019). Consequently, plants can significantly influence the restructuring of the resident soil microbiome to establish a healthy rhizosphere microbial community. However, the resident soil microbial community may resist this restructuring, maintaining its composition due to strong resilience against biotic and abiotic perturbations (Lourenço et al., 2018a). Furthermore, this resilience can also reverse desired functional changes resulting from microbial amendments even after repeated inoculations (Zhikang et al., 2021a).

The resident soil microbiome accounts for a large proportion of the rhizosphere microbiome (Chen et al., 2023a; Xun et al., 2024), which functions as a primary line of defense for plant immunity (Carrión et al., 2019). This line of defense could deter the establishment of phytopathogens while also hindering beneficial microbes intended to control pathogens (Russ et al., 2023). Such an effect was observed for *Pseudomonas aeruginosa*, a pathogen-antagonistic bioinoculant, whose population declined by 99% within one week of inoculation, indicating that the microbial line of defense exerted strong inhibitory pressure that prevented its establishment (Thomas and Sekhar, 2016). Manure-borne microbes were also found to have failed to establish in the rhizosphere due to intense competitive interactions, which led to them being outcompeted (Schlatter et al., 2022), indicating that the benefits to plants arise more from manure-derived nutrients than from manure-associated microbes.

Studies focusing on relative abundance metrices have provided important insights (Lourenço et al., 2018b; Zhikang et al., 2021b; Xun et al., 2023; Rasool et al., 2025a) in explaining the role of a resident soil microbiome on the fate of microbial inoculants. However, the influence of overall microbial load, fundamental for understanding microbial community dynamics (Vandeputte et al., 2017), has remained largely unexplored. Furthermore, most previous studies have concentrated on the impacts of specific microbial taxa or synthetic communities, such as plant growth–promoting rhizobacteria (PGPR), mycorrhizal fungi, or biocontrol agents, typically linking their effects to localized changes in plant defense or metabolism. As a result, the broader system-level consequences of microbiome restructuring on host transcriptomic regulation remain poorly understood. To address these gaps, we investigated how pre-planting modulation of resident soil microbial load influences rhizosphere restructuring and plant health. Specifically, we examined how quantitative microbiome dynamics regulate the efficacy of microbial inoculation and cucumber transcriptomic responses. We hypothesized that inducing dysbiosis in the resident soil microbiome would alter microbial load and diversity, thereby enhancing inoculation efficacy and enabling *Cucumis sativus* L. to restructure its rhizosphere microbiome toward a healthier state. To investigate this, we established an experimental gradient of microbial diversity by inducing dysbiosis in the resident soil microbiota through MHT. Dysbiosis is typically associated with reduced host fitness (Levy et al., 2017; Arnault et al., 2023). However, unlike host-associated microbiomes, resident soil microbial communities exist independently of direct host regulation. In this context, we conceptualized dysbiosis in the resident soil microbiome as a substantial shift in both the relative and absolute abundance of microbial communities, accompanied by functional alterations.

We evaluated the effects of dysbiosis and microbial inoculation by integrating phenotypic assessments of *Cucumis sativus*, quantitative and relative profiling of the rhizosphere microbiome, and transcriptomic analysis using DESeq2. Our results emphasize the central role of microbial load modulation in shaping plant health and determining inoculation efficacy, thereby offering potential strategies for optimizing rhizosphere microbiome function.

## Materials and methods

### Soil sampling and experimental setup for amplicon sequencing

A soil sample was collected in May 2023 from the top 15 cm of a plot at the agricultural research greenhouse at Kyungpook National University (KNU) Daegu campus (35°53′41″N, 128°36′49″E, 36.17 m above sea level), which has a history of pathogenic incidence in cucumber (*Cucumis sativus*) cultivation. The sample was then taken to the laboratory and sieved through an 8 mm mesh to remove large roots and stones. To improve moisture retention, the soil was then homogenized by blending it in a 1:1 ratio with potting soil that had been autoclaved three times at 115°C for 45minutes to eliminate potential microbial contamination. The soil was subsequently moistened with sterile water, thoroughly mixed to ensure uniform moisture distribution, and divided into two sets. The first set was subjected to moist heat treatment (MHT) at 105 °C for 15 min, while the second set was used for planting without MHT. After MHT, the soil was kept at room temperature for 3 days before sampling to perform chemical analysis and DNA extraction for pretreatment microbial quantification.

### Planting material preparation

Cucumber seeds (cv. Asiamiin) were obtained from Asia Seed Company (Seoul, South Korea). The seeds were gently washed with sterile water. The surface of each sample was disinfected by treatment with 70% ethanol for 3 min, followed by incubation with 0.5% sodium hypochlorite solution for 7 min. The samples were then rinsed four times with sterile water. To confirm effective surface disinfection, the final wash was plated onto potato dextrose agar (PDA) and nutrient agar and incubated at 25 °C and 30 °C, respectively, for three 3 days. The absence of microbial growth on either medium throughout the incubation period confirmed the successful elimination of surface contaminants.

### Bioagent inoculum preparation

The bioagent *Fusarium* sp. isolate RH2 (KNU, Laboratory of Molecular Microbiology) exhibited *in vitro* antagonistic activity against *Alternaria* sp. and Sclerotinia sp. pathogens isolated from the cucumber rhizosphere. The pathogen *S. sclerotiorum* (Lib.) KF1 (KNU, Laboratory of Molecular Microbiology) was specifically isolated from diseased cucumber plant. A mycelial plug of the *Fusarium* isolate was cultured on a fresh PDA plate and incubated at 26 °C. After 3 days, actively growing mycelial margins were transferred to potato dextrose broth (PDB) and incubated in a shaking incubator at 26 °C with continuous shaking at 150 rpm for 2 weeks. The resultant mycelial suspension was filtered through sterile filter paper (nominal retention: 5 µm) to separate the microconidia from the mycelial plugs. The filtered microconidia were subsequently resuspended in sterile water to eliminate any remaining culture medium, quantified via a hemocytometer (Thermo Fisher Scientific, Waltham, MA, USA), and applied as a drench at a rate of 1 × 10^7^conidia mL^−1^ to the planting spot immediately after the cucumber seeds were planted.

### Pathogen inoculum preparation

A sclerotium (~2 mm diameter) from a pure culture of *S. sclerotiorum* KF1 was transferred to a fresh PDA plate and incubated at 25 °C. After 3 days, actively growing mycelial margins were excised and inoculated into PDB, followed by incubation in a shaking incubator at 25 °C with shaking at 150 rpm for one week. The resulting mycelial suspension was homogenized with sterile glass beads (0.5 mm diameter) at low speed for 60 s, effectively fragmenting the mycelia into smaller pieces to promote uniform distribution in the suspension. The mycelial fragments were then filtered and resuspended in sterile water to remove the culture medium. Subsequently, the number of mycelial fragments in the suspension was quantified using a hemocytometer (Thermo Fisher Scientific, Waltham, MA, USA), and a 1 mL aliquot of the homogenized suspension (approximately 10^7^ fragments mL^−1^) was drenched onto the planting spot after seeds were planted.

### Experimental design

The first experiment was designed in two sets: one with moist heat treated (dysbiotic) soil and the other with moist heat untreated (native) soil. Each set consisted of four treatments (three types of microbial inoculations, namely, a bioagent, a bioagent + a pathogen, and a pathogen, and an untreated control), with eight replications per treatment.

Nursery tray cells with a well volume of 150 cm³ were used as an experimental unit. Nine wells, organized into three rows, with each well containing a drainage hole at the bottom and sharing a common base plate, were filled with soil following the experimental design. Eight sets of wells were established as replicates, with each set receiving the same treatment, except for the central well, which remained unplanted to ensure a uniform environment across replicates and improve aeration.

Two surface-disinfected seeds were planted in each well and subjected to the designated microbial treatments as per the experimental layout. A 1 mL aliquot of the microbial treatment suspension was subsequently applied as a soil drench to each well at the planting location. To maintain uniform conditions, the untreated control was drenched with sterile water. The microconidia suspension of the bioagent and the mycelial suspension of the pathogen were directly applied as soil drenches after planting, and the seeds were covered with a thin film of soil.

### Soil chemical analysis

Soil samples from both native and dysbiotic sets were analyzed in triplicates for their chemical properties at the Soil Physical and Chemical Analysis Laboratory of the Korean Agricultural Technology Promotion Agency. The mean values of the analyzed soil chemical parameters: pH (measured in a 1:5 soil:water suspension), total nitrogen content (%), total organic carbon content (%), total phosphorus content (mg/kg), exchangeable potassium content (cmolc/kg), and iron content (%) were presented in **Supplementary File: Table S1**.

### Experimental design for transcriptomic analysis

The second experiment was conducted to assess the specific effects of bioagent inoculation on cucumber (*Cucumis sativus* L.) seedlings grown in dysbiotic soil. Soil from the same homogenized sample was collected and prepared as described in the “Soil sampling and experimental setup” section, adjusted to uniform moisture content, and divided into two groups (bioagent-treated and untreated control), each comprising three replicate pots (top diameter: 80 mm, bottom diameter: 75 mm, height: 75 mm; volume: 353.4 mL). Three pots per group, containing the respective soils, were placed in separate 5,000-mL microbox containers (base: 185 × 185 mm; lid: 195 × 195 mm), sealed, and subjected to moist-heat treatment at 105 °C for 15 min to induce dysbiosis. Following a 3-day incubation period, surface-sterilized cucumber seeds were aseptically sown in the pots within a laminar flow hood. One group received post-planting bioagent inoculation, while the other served as an untreated control. Microboxes were resealed and maintained under controlled conditions for 30 days to exclude confounding environmental microbial influences, thereby isolating the bioagent’s specific effects on the plant’s transcriptomic response. On day 30, actively growing shoot apices were harvested from three replicate plants per treatment, immediately flash-frozen in liquid nitrogen, ground to a fine powder, and stored at −80 °C pending RNA extraction.

### Sampling and data collection

Soil samples for genomic DNA (gDNA) extraction before planting (n = 10) were collected from five randomly selected plots in both native and dysbiotic sets. Rhizosphere soil samples (n = 40) were obtained 30 days after planting from five randomly selected plots in both sets. The seedlings were carefully uprooted, and loosely attached soil was then removed by thorough shaking. The soil that was firmly adhered to the roots was collected as rhizosphere soil for further processing. In plots where all the seedlings had died, samples were taken directly from the planting wells after the topsoil layer was removed.

Seedling germination and survival were monitored daily for 30 days. Phenotypic data regarding stem thickness and chlorophyll content were recorded for every surviving plant across all replications 30 days after planting. The chlorophyll content was measured using a chlorophyll meter (SPAD-502Plus, Konica Minolta, Tokyo, Japan), and stem thickness was recorded using a digital caliper (SD-500-150PRO, SINCON, Seoul, Korea). The mean of the measurements from each replication was used as the representative value. The soil and plant samples were separately collected in sterile screw cap centrifuge tubes and stored at −80 °C until DNA extraction.

### DNA extraction and amplicon sequence library preparation

gDNA was extracted from homogenized 0.25 g soil samples (n = 50) via the DNeasy PowerSoil^®^ Pro Kit (Qiagen, Hilden, Germany) following the manufacturer’s instructions. All the wash and elution steps were performed as instructed, with a final elution volume of 40 μL for the dysbiotic rhizosphere samples and 50 μL for the native rhizosphere samples. DNA concentrations were quantified via a Qubit^®^ 2.0 fluorometer (Thermo Fisher Scientific, Waltham, MA, USA) and normalized to 5 ng/μL. Given the low concentration of gDNA extracted from dysbiotic soil samples before planting (n = 5), the full-length 16S ribosomal RNA (rRNA) gene was first amplified via the primer pair 27F (5′-AGAGTTTGATCMTGGCTCAG-3′) and 1492R (5′-TACGGYTACCTTGTTACGACTT-3′) (Lane, 1991). The resulting PCR product was then used as a template for subsequent amplification during Illumina MiSeq library preparation.

### Amplicon sequencing and bioinformatics

Amplicon sequencing was performed via the Illumina MiSeq platform (Illumina Inc., San Diego, CA, USA), which targets the V4 region of the 16S rRNA gene. Amplification was performed via the primer pair 515F (5′-GTGCCAGCMGCCGCGGTAA-3′) and 806R (5′-GGACTACHVGGGTWTCTAAT-3′) (Caporaso et al., 2011; Bates et al., 2011). The PCR amplicons were purified via AMPure XP magnetic beads (Beckman Coulter, Brea, CA, USA). A second PCR step was then performed to attach Illumina sequencing adapters and dual indices via the Nextera^®^ XT Index Kit (Illumina, San Diego, CA, USA) according to the manufacturer’s instructions. The indexed PCR products were again purified with AMPure XP beads and quantified via a Qubit 2.0 fluorometer, and their concentrations were normalized before sequencing. Library quality, fragment size distribution, and concentration were assessed via an Agilent 2100 bioanalyzer (Agilent Technologies, Santa Clara, CA, USA). The final amplicon library was pooled in equimolar concentrations and quantified via a Qubit 2.0 fluorometer. Sequencing was performed via the MiSeq Reagent Kit v. 2 (300 bp single-end) on the Illumina MiSeq platform at the NGS Core Facility, KNU, Daegu, South Korea.

Paired-end molecular sequence data were processed via QIIME 2 v. 2024.10 (Bolyen et al., 2019). Denoising was performed via the DADA2 algorithm (Callahan et al., 2016), and reads were truncated at 299 bp and quality filtered, with a maximum expected error of 0.5. After removing replicates and singletons, the remaining reads were assigned to amplicon sequence variants (ASVs) at a 99% identity threshold. Taxonomic classification of representative sequences was performed via the SILVA 138-99-nb diverse-weighted classifier (Kaehler et al., 2019). The Scikit-learn machine learning algorithm (Pedregosa et al., 2011) was then used to assign taxonomy to ASVs. Multiple sequence alignment was performed via MAFFT (Katoh and Standley, 2013), and low-confidence regions were masked before phylogenetic tree construction. Rooted and unrooted phylogenetic trees were constructed via the FastTree algorithm (Price et al., 2010). The generated taxonomy-assigned contaminants, including chloroplasts, mitochondria, and unclassified kingdom-level taxa, were excluded from downstream analyses in QIIME 2 environment.

### RNA extraction, library preparation, and transcriptome sequencing

Total RNA was extracted from lyophilized cucumber (*Cucumis sativus* L.) leaf samples via the DNeasy Plant Mini Kit (Qiagen, Hilden, Germany) according to the manufacturer’s protocol. RNA integrity was evaluated via an Agilent 2100 Bioanalyzer (Agilent Technologies, Santa Clara, CA, USA), and samples with an RNA integrity number (RIN) ≥ 6.5 were selected for downstream analysis. The RNA sequencing libraries were constructed via the MGIEasy RNA Library Prep Set (MGI Tech Co., Ltd., Shenzhen, China) following the manufacturer’s instructions. Sequencing was performed at the KNU NGS Core Facility via the DNBSEQ-G400 platform (MGI Tech), generating 100-bp paired-end reads. On average, each sample yielded approximately 38.79 million raw paired-end reads. Adapter trimming and quality filtering were conducted via Trimmomatic v0.39 (Bolger et al., 2014) with the parameters ILLUMINACLIP: TruSeq3-PE.fa:2:30:10, LEADING:3, TRAILING:3, SLIDINGWINDOW:5:30, and MINLEN:30.

### Statistical analysis of microbiome and phenotypic data

The statistical analysis of microbiome data was performed in the R environment (v. 4.4.3) via several packages, including phyloseq version 1.46.0 (McMurdie and Holmes, 2013), dplyr version 1.1.4 (Wickham et al., 2023), vegan Version 2.6-10 (Oksanen et al., 2025), ggplot2 version 3.5.1 (Wickham et al., 2016), tidyverse version 2.0.0 (Wickham et al., 2019), FSA version 0.10.0 (Ogle et al., 2025), ggalluvial version 0.12.5 (Brunson, 2020), and tidyr version 1.3.1 (Wickham et al., 2024). Alpha diversity metrics were calculated with QIIME2 and visualized in R software via the Kruskal–Wallis test for assessing species richness (Chao1 index), diversity (Shannon and Simpson indices), and evenness (Pielou’s index). Principal coordinate analysis (PCoA) with the Bray–Curtis dissimilarity index was used to compare microbial compositions between the dysbiotic rhizospheres and native rhizospheres, and among the microbial inoculations and the control. The patterns elucidated by PCoA were statistically compared via the adonis (permutational multivariate analysis of variance [PERMANOVA]) function in the vegan package with 999 permutations. To ensure reproducibility, the seed value (set.seed(123)) was specified prior to all analyses. The bacterial co-occurrence network was constructed using the WGCNA package in R (Langfelder and Horvath, 2008), where edges were initially determined from adjacency matrices generated by WGCNA, followed by the application of a correlation threshold of r > 0.7 (Langfelder and Horvath, 2008). The network modules were subsequently identified via the fast greedy clustering algorithm (Clauset et al., 2004). The resulting network was exported in GEXF format and visualized via Gephi v. 0.10.0 (Bastian et al., 2009). Core genera were identified via the LEfSe method implemented in the microeco package (Segata et al., 2011; Liu et al., 2021a), which applies the Kruskal–Wallis test to detect differentially abundant taxa across treatments, followed by linear discriminant analysis (LDA) to estimate effect sizes (α ≤ 0.05). Redundancy analysis (RDA) was performed to assess relationships among MHTs, microbial inoculations, microbial compositions, and phenotypic responses. Phenotypic data were analyzed via ANOVA after normality was assessed via the Shapiro–Wilk test and variance homogeneity via Levene’s test; Tukey’s HSD test was used for pairwise comparisons.

Structural equation modeling (SEM) was employed to evaluate the causal pathways linking phenotypic responses, germination percentage and seedling survival to MHT, absolute microbial abundance, relative microbial diversity, and microbial inoculation treatments. Data normality was assessed via the Shapiro–Wilk test, and graphical visualization of the SEM results was conducted in R via the lavaan package version 0.6-20 (Rosseel, 2012), and semPlot version 1.1.6 (Epskamp, 2015) packages.

### Functional profile predictions

PICRUSt2 (Douglas et al., 2020) v. 2.6.0 was used to examine the potential functional profiles of the bacterial communities in the rhizosphere before and after MHT with the respective microbial inoculations. The EC number, KO metagenome, and MetaCyc pathway abundance were predicted on the basis of the inferred EC number abundance data via the representative sequences and the BIOM table of ASV abundances across samples as input. The nearest sequenced taxon index (NSTI) was calculated for each input ASV, and any ASVs with NSTI > 2 were excluded from the output by default.

The R package ggpicrust2 (Yang et al., 2023) was used to analyze and visualize functional profile predictions from PICRUSt2. MetaCyc pathway abundance tables from PICRUSt2 were used as inputs to perform differential abundance analysis to compare functional profile differences between the dysbiotic and native rhizospheres and their respective microbial inoculations treatments. Functionally enriched pathways were analyzed via LinDA (Zhou et al., 2022), and the results were annotated without KO-to-KEGG conversion. The results were visualized via principal component analysis plots, pathway heatmaps, and pathway error bars.

### Quantitative microbiome profiling via droplet digital polymerase chain reaction

Absolute quantification of bacterial communities in the rhizosphere was performed via ddPCR with a reaction mixture volume of 20 μL. Droplets were then generated via a droplet generator (DG) with 70 μL of DG oil/well, along with a DG8 cartridge and cartridge holder, 20 μL of PCR mixture, and a DG8 gasket. The droplets were dispensed into a 96-well PCR plate by aspirating 40 μL from the DG8 cartridge into each well. The PCR plate was then heat sealed with a foil seal and placed in a thermocycler (C1000, Bio-Rad). The PCR protocol consisted of an initial cycle at 95°C for 5 min, 40 cycles at 95°C for 30 s and 57°C for 1 min, and a final cycle at 4 °C for 5 min and 90 °C for 5 min with a 4°C hold. The droplets were subsequently analyzed via a droplet reader, and the data were processed via QuantaSoft software. The 16S rRNA gene copy numbers per gram of soil were then calculated, and the absolute abundance of microbial taxa was estimated on the basis of their relative abundance. To account for variations in rRNA operon copy numbers across bacterial taxa, absolute abundance was normalized via rrnDB (Stoddard et al., 2015). The normalized absolute counts were visualized via the ggplot2 package in R (Wickham et al., 2016). Absolute abundance data were compared via the Kruskal–Wallis test and Wilcoxon pairwise comparison test to determine the statistical significance of changes in the absolute abundance of bacterial communities.

### Differential gene expression and functional analysis

Transcript abundance was quantified from RNA-Seq reads aligned to the *Cucumis sativus* reference genome (GCF_000004075.3_Cucumber_9930_V3) via StringTie v2.2.3 (Pertea et al., 2015). Gene expression, estimated in FPKM, was compiled into matrices for control and bioagent-treated samples. Differential gene expression (DGE) analysis was performed via DESeq2 (Love et al., 2014) to compare conditions, generate a volcano plot, and identify genes of interest. Genes with p values ≤ 0.05 and |log2 FC| values ≥ 1 were classified as DEGs. The DEGs annotated with GO terms and enrichment results were visualized via ggplot2 (Wickham et al., 2016). PCA was used to assess sample variability, and a hierarchical clustering heatmap was used to illustrate distinct DEG expression patterns between bioagent-treated and control samples.

## Results

### Characterization of moist heat induced dysbiosis in the resident soil microbiome

The impact of MHT on the alpha diversity of the resident microbiome showed significant differences in Simpson’s and Pielou’s indices (p < 0.05 and p < 0.01, respectively (**Supplementary File: Figure S1A**). This indicates that MHT resulted in uneven microbial diversity in the dysbiotic rhizosphere compared with the native one. PCoA and PERMANOVA (**Supplementary File: Figure S1B**) revealed significant differences in microbial community composition between dysbiotic and native soils (PERMANOVA, R² = 0.114, p < 0.01). Pairwise Bray–Curtis dissimilarity comparisons further confirmed this distinction, indicating highly significant compositional differences between the dysbiotic and native rhizospheres (p < 0.001; **Supplementary File: Figure S1C**).

Quantitative microbiome profiling revealed that MHT significantly reduced the absolute bacterial abundance within the resident soil microbiota (Wilcoxon, p < 0.01), causing an average decrease of 96.4% ± 0.9%. (**Supplementary File: Figure S1D**, **Supplementary File: Table S2**). Moreover, MHT induced major shifts in the microbial community composition, causing complete depletion of the phylum Crenarchaeota and a significant decline in Actinobacteria (Wilcoxon, *p* < 0.01) in the treated soils (**Supplementary File: Supplementary Figure S1D**, **Supplementary Table S3**). Conversely, following MHT, the relative abundance of Firmicutes increased significantly (Wilcoxon test, *p* < 0.05), likely due to the displacement of other phyla. These compositional shifts led to broader restructuring among prokaryotic phyla before and after MHT, with potential functional consequences.

The functional predictions revealed significant deviations in the pathway abundance from the mean, a significant divergence in the predicted metabolic pathways in the PCoA, and highly significant log2-fold changes between the dysbiotic and native soils (**Supplementary File: Figure S2A-C**, respectively), indicating significant functional shifts in the microbial communities. In native soil, predicted metabolic pathways related to carbohydrate metabolism, aromatic compound biosynthesis, organic acid and small molecule degradation, and cofactor biosynthesis were enriched while these pathways were significantly depleted in dysbiotic soil. In contrast, pathways such as coenzyme B biosynthesis, nitrate reduction VI (assimilatory), coenzyme M biosynthesis I, and L-valine degradation have reduced abundance, indicating a decline in their metabolic activity under untreated condition. (**Supplementary File: SupplementaryFigure S2C**). Despite these significant alterations in the resident soil microbiota structure, no substantial changes were noted in the soil chemical properties after MHT (**Supplementary File: Supplementary Table S1**).

### Planting promotes substantial enrichment of the rhizosphere microbiome after MHT

**Figure 1:**
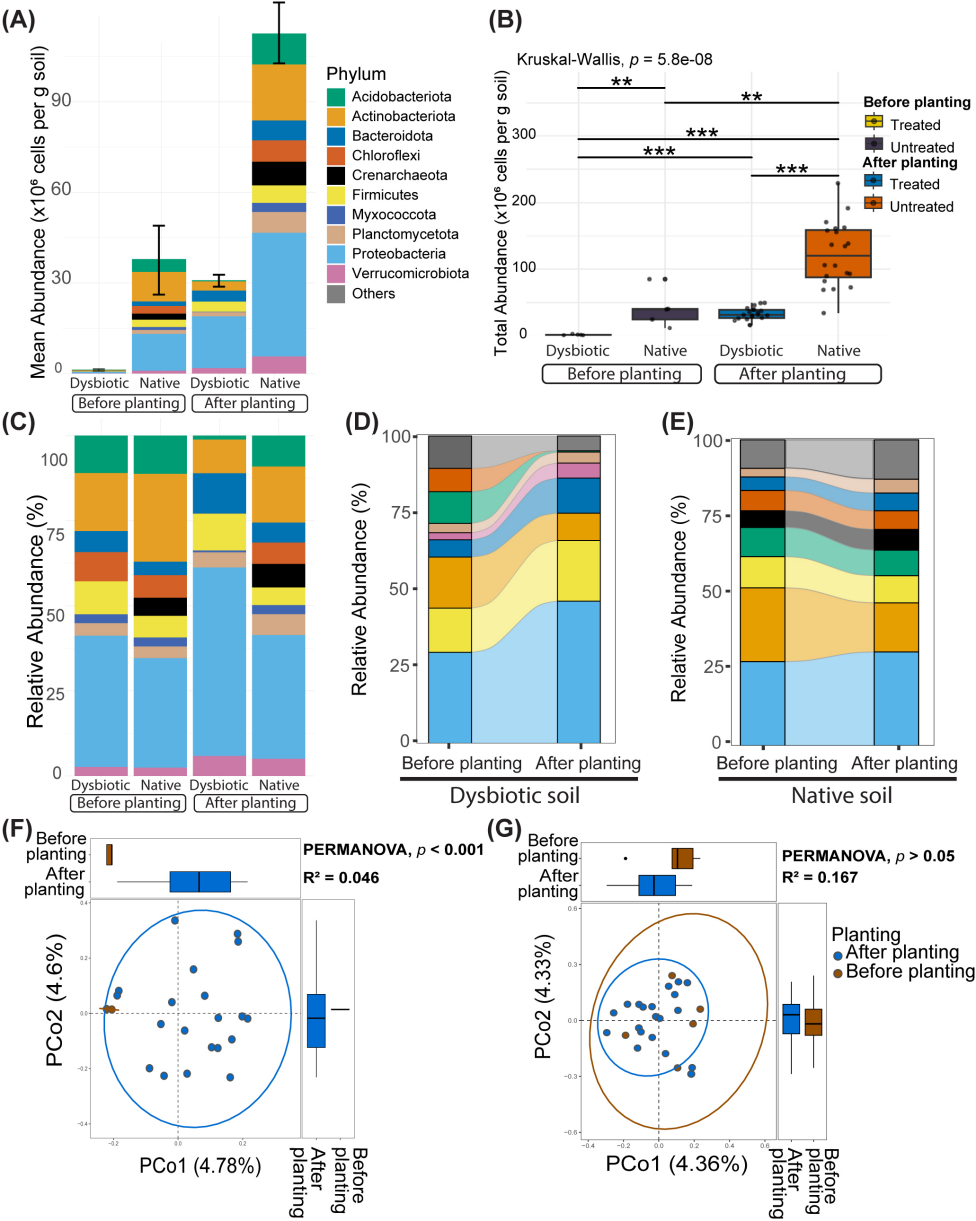
Impact of cucumber planting on the restoration of bacterial abundance and compositional shifts **(A)** Changes in the absolute abundance of prokaryotic phyla in dysbiotic and native soils before and after planting. **(B)** Kruskal–Wallis test results and Wilcoxon pairwise comparisons of mean absolute abundance data before and after cucumber planting, with significant differences indicated by ** (*p* < 0.01) and *** (*p* < 0.001). **(C–E)** Relative abundance data of the top ten prokaryotic phyla, illustrating compositional differences in dysbiotic and native soils before and after planting. **(F, G)** Beta diversity PCoA shows **(F)** significant shifts in bacterial community composition before and after planting in dysbiotic soil and **(G)** nonsignificant differences before and after planting in native soil.

Quantitative microbiome profiling revealed recovery of the microbial abundance that had been disrupted by moist heat-induced dysbiosis from 3.6% to 81.6%, whereas in the native rhizosphere, a threefold increase in the quantitative bacterial abundance (QA) was observed (**Figure 1A**, **Supplementary File: Table S2**). The Kruskal–Wallis test indicated a highly significant increase in absolute microbial abundance in both dysbiotic and native rhizospheres (p < 0.001; **Figure 1B**). Pairwise comparisons via the Wilcoxon rank-sum test indicated that the recovered microbial abundance after planting in the dysbiotic rhizosphere was statistically similar to the microbial abundance observed in the native soil before planting (p > 0.05; **Figure 1B**, **Supplementary File: Table S2**).

In the dysbiotic rhizosphere, the restoration of microbial abundance was accompanied by a significant shift in microbiome composition and structure (PERMANOVA, p < 0.001; **Figures 1C, D, F**). In contrast, in the native rhizosphere, no significant shift in microbial community composition was observed after cucumber planting (PERMANOVA, p > 0.05; **Figure 1C, E, G**). Principal Coordinates Analysis (PCoA) of beta diversity showed that the native rhizosphere maintained a consistent microbial community composition, closely aligning with the preplanting soil microbiome (**Figure 1G**). In contrast, the dysbiotic rhizosphere displayed significant shifts in microbial community composition post-planting, with greater beta diversity than the native rhizosphere (**Figure 1F**).

### The native soil microbiome confers resistance to inoculation-induced changes

**Figure 2:**
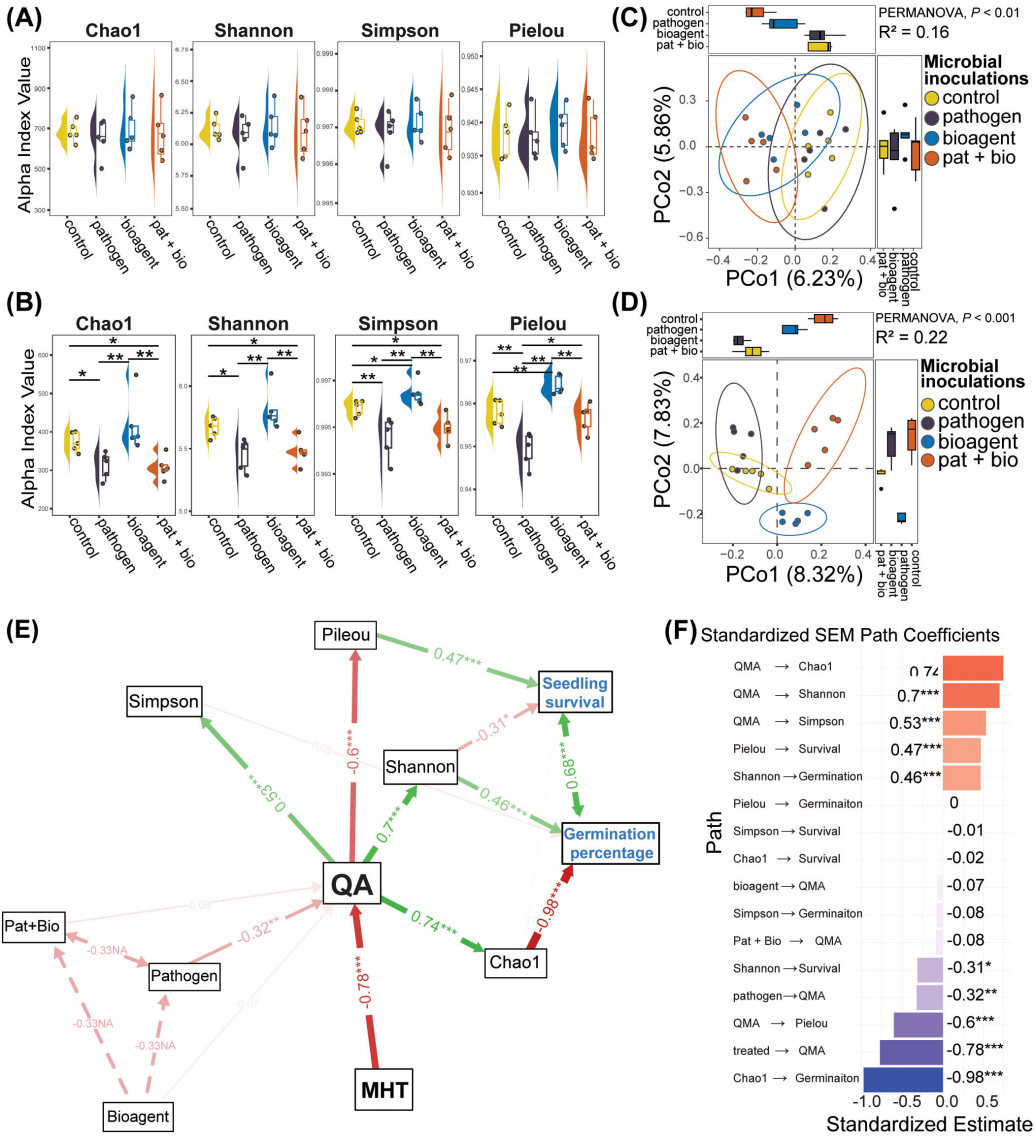
Analysis of prokaryotic microbiome diversity in native and dysbiotic rhizospheres. **(A)** Alpha diversity metrics show no statistical differences across microbial inoculation treatments: untreated control, pathogen, bioagent, and pathogen + bioagent (pat + bio) in the native rhizosphere. **(B)** Significant statistical differences in alpha diversity metrices across microbial inoculation treatments in the dysbiotic rhizosphere. **(C, D)** PCoA of beta diversity distances reveals marked changes in bacterial community structure across microbial inoculation treatments in both **(C)** native and **(D)** dysbiotic rhizospheres. **(E)** SEM depicting effects of MHT and microbial inoculations on cucumber germination percentage and seedling survival. MHT reduces QA, resulting in lower evenness (Pielou’s index), increased richness and a decreased Shannon index. This collectively improves germination and survival of cucumber. Solid and dashed arrows represent significant and nonsignificant relationships between measured variables, respectively, while green and red arrows represent positive and negative associations, respectively. Arrow width reflects the strength of path coefficients. *, **, and *** denote significance levels of p < 0.05, 0.01, and 0.001, respectively. **(F)** standardized SEM path Coefficients showing the standardized estimates and their significance.

The application of microbial inoculants (including a bioagent, a pathogen, or their combination) and an untreated control resulted in distinct responses in native and dysbiotic rhizospheres. In the native rhizosphere, microbial inoculants exhibited statistically identical responses to those of the untreated control (p > 0.05; **Figure 2A**) across the richness and evenness metrics of alpha diversity. However, in the dysbiotic rhizosphere, microbial inoculants led to significant differences in alpha diversity compared with the untreated control across all alpha diversity indices (p < 0.05; **Figure 2B**). Pathogen inoculation and combined pathogen + bioagent inoculation led to significantly lower Chao1 and Shannon diversity values than did the control or bioagent-only inoculation. Additionally, bioagent-treated rhizospheres exhibited significantly higher Shannon, Simpson, and Pielou indices, indicating greater microbial diversity and evenness compared to the control and other inoculation groups (**Figure 2B**).

Beta diversity among microbial inoculations in both native and dysbiotic rhizospheres was statistically significant, yet the magnitude of the structural shift was smaller in the native rhizosphere (PERMANOVA, p< 0.01; R^2^ = 0.16; **Figure 2C**) than in the dysbiotic rhizosphere (PERMANOVA, p < 0.001; R^2^ = 0.22; **Figure 2D**).

SEM revealed that QA was a central variable that was significantly negatively affected by MHT (**Figure 2E**). This reduction in QA predicted shifts in microbial diversity, which in turn influenced the cucumber seeds germination percentage and seedling survival. Specifically, the MHT-induced reduction in bacterial QA led to a decrease in Chao1 diversity, which emerged as a dominant and significant positive predictor of germination percentage. Conversely, the reduction in QA was associated with an increase in Pielou’s evenness, which significantly enhanced seedling survival. Among the microbial treatments, pathogen application was significantly negatively associated with QA. Notably, while decreased Shannon diversity, resulting from reduced QA, was linked to lower germination percentages, it concurrently promoted greater seedling survival (**Figure 2E, F**).

**Figure 3:**
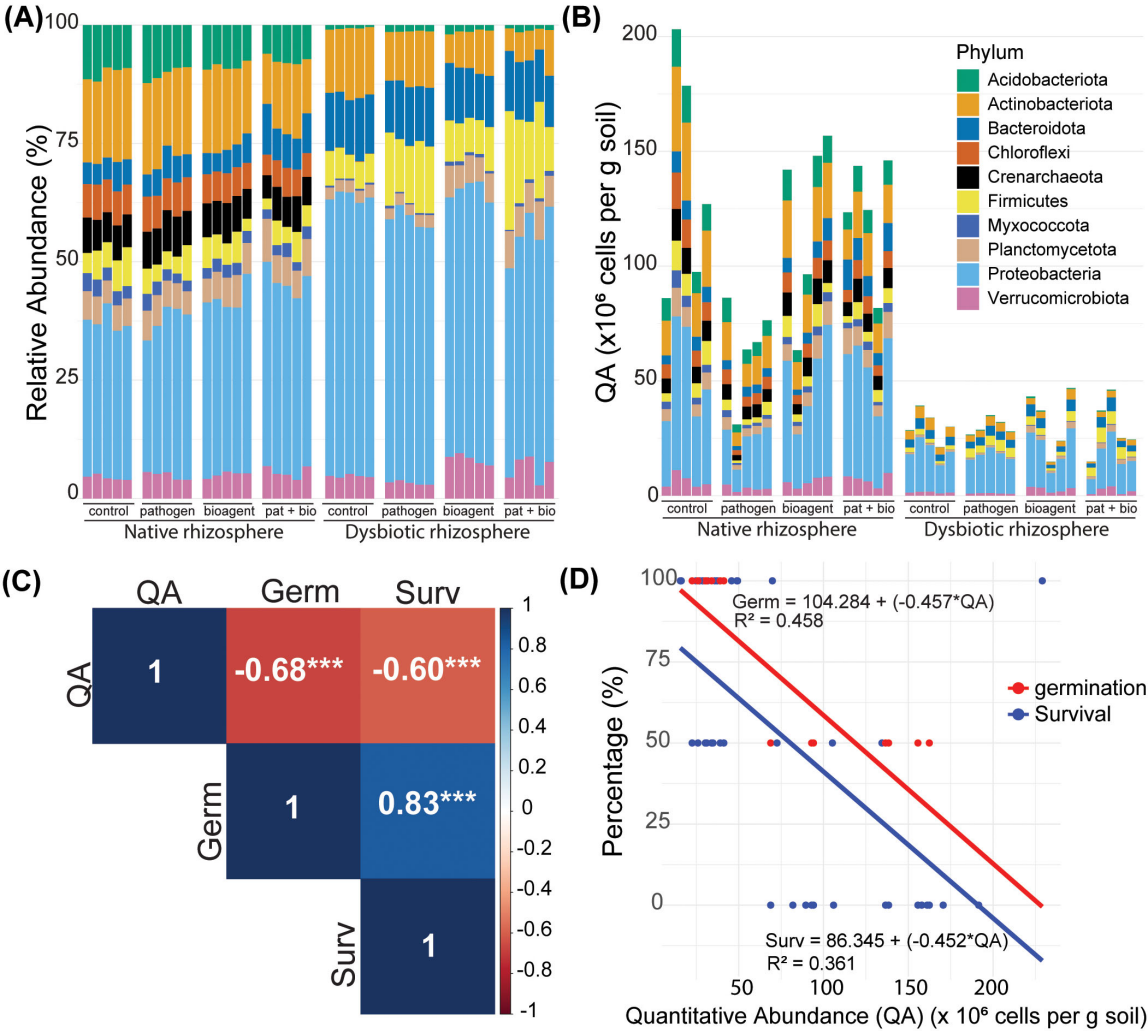
Impact of QA on seed germination and seedling survival in *Cucumis sativus* L. **(A)** Relative abundance of the ten most dominant prokaryotic phyla under native and dysbiotic rhizosphere conditions, showing distinct differences in microbial community composition across treatments. **(B)** Total QA was markedly reduced in the dysbiotic rhizosphere compared to the native condition. **(C)** QA was negatively correlated with both seed germination (Germ) and seedling survival (Surv), while germination and survival were positively correlated. *** indicates *p* < 0.001. **(D)** Linear regression analysis depicted that increasing QA was associated with progressive reductions in germination and survival rates, explaining 45.8% and 36.1% of the variance, respectively.

Quantitative microbiome profiling revealed significantly lower QA in the dysbiotic rhizosphere than in the native rhizosphere (**Figure 3B**; Kruskal–Wallis, p < 0.001; **Supplementary File: Figure S3**). Furthermore, the relative abundances of the top eight prokaryotic phyla differed significantly between the native and dysbiotic rhizospheres (Wilcoxon test, *p* < 0.001; **Supplementary File: Supplementary Table S4**). The dysbiotic rhizosphere, characterized by lower QA, exhibited statistically similar absolute abundances (p > 0.05; **Supplementary File: Figure S3**, **Supplementary Table S5**) but showed significant compositional shifts among microbial inoculation treatments across the top nine prokaryotic phyla (**Supplementary File: Table S6**). These shifts were primarily driven by increased relative abundances of Proteobacteria, Firmicutes, and Bacteroidota (**Figure 3A**; **Supplementary File:Supplementary Table S7**). In contrast, the native rhizosphere displayed significant compositional changes among microbial treatments across the top seven prokaryotic phyla, with Firmicutes showing no significant differences among treatments (**Supplementary File: Tables S8, S9**). The native rhizosphere was instead enriched in Acidobacteriota, Actinobacteriota, and Chloroflexi (**Figure 3A**).

Correlation analysis revealed significant negative correlations of QA with the germination percentage (Pearson’s r = -0.68) and seedling survival (Pearson’s r = -0.60) of *Cucumis sativus* L., with a strong positive correlation observed between the germination percentage and seedling survival (**Figure 3C**). Linear regression analyses further elucidated these relationships, with germination percentage modeled as Germ = 104.284 - 0.457QA (R² = 0.458) and seedling survival as Surv = 86.345 - 0.452QA (R² = 0.361), indicating that quantitative bacterial abundance accounted for 45.8% of the variation in germination percentage and 36.1% of the variation in seedling survival (**Figure 3D**).

### A decline in the co-occurrence network underscores shifts in the rhizosphere microbiome

**Figure 4:**
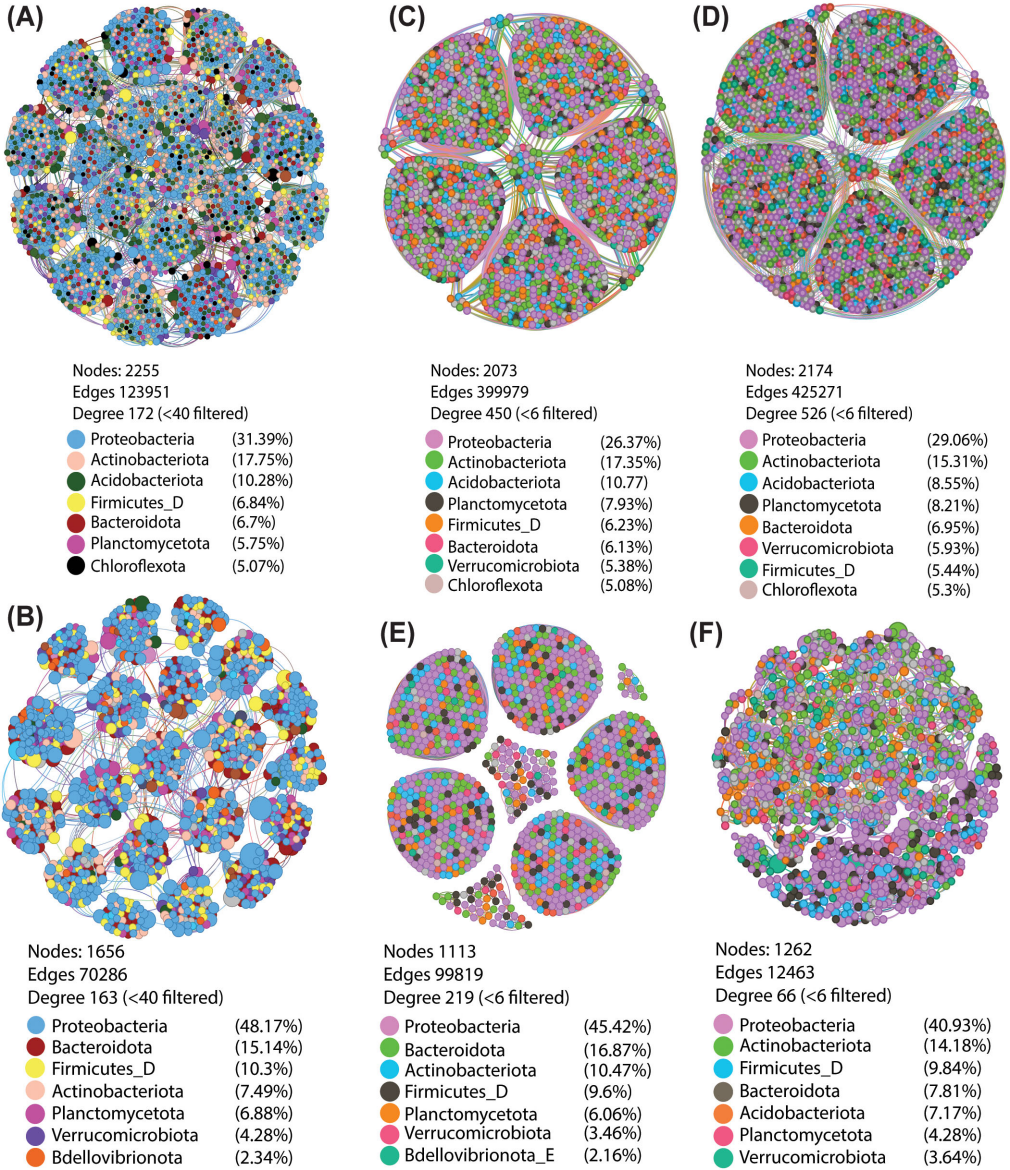
Bacterial co-occurrence networks illustrating bacterial community connectivity in the rhizospheres of cucumber seedlings. **(A)** Native rhizosphere displaying a dense, highly interconnected bacterial network. **(B)** Dysbiotic rhizosphere exhibiting a simplified network structure with distinct compositional changes. **(C)** Native rhizosphere without microbial inoculation (untreated control) and **(D)** native rhizosphere with bioagent inoculation, both showing consistent network patterns. **(E)** Dysbiotic rhizosphere without microbial inoculation and **(F)** dysbiotic rhizosphere with bioagent inoculation, revealing distinct network configurations and shifts in keystone taxa. Node size represents betweenness and centrality, with larger circles indicating higher betweenness and centrality, and node colors denote specific bacterial phyla.

Bacterial co-occurrence network analysis of the native rhizosphere revealed a highly intricate and interconnected structure comprising 2,255 nodes and 123,951 edges, with an average degree of 172 (**Figure 4A**). In contrast, the dysbiotic rhizosphere presented significantly reduced network complexity, as evidenced by a decrease to 1,656 nodes and 70,286 edges, with a slightly lower average degree of 163 (**Figure 4B**). This structural simplification was accompanied by substantial taxonomic reorganization, where Bacteroidota and Firmicutes supplanted Actinobacteriota and Acidobacteriota as the second and third most dominant phyla, respectively, alongside an approximately 18% increase in the relative abundance of Proteobacteria (**Figure 4B**). MHT-induced dysbiosis in uninoculated rhizospheres resulted in a marked reduction in network complexity, with 46% loss of nodes and 75% loss of edges, leading to an overall 51% decrease in network connectivity compared with that in the native rhizosphere (**Figure 4C, E**). The complex network in the native rhizosphere, dominated by Proteobacteria, followed by Actinobacteria and Acidobacteria, likely reflects a functionally stable and resilient microbial community in the absence of MHT (**Figure 4A**).

In the native rhizosphere, the bacterial co-occurrence networks under bioagent-treated and untreated conditions presented comparable structural and compositional patterns (**Figure 4C, D**). Conversely, bioagent treatment in the dysbiotic rhizosphere induced a significant reduction in network connectivity, with the average degree decreasing from 219–66 and edges decreasing from 99,819–12,463, while node count remained comparable (**Figure 4E, F**). Additionally, bioagent treatment in the dysbiotic rhizosphere led to a reorganization of keystone taxa (**Figure 4F**).

### MHT caused significant shifts in the predicted rhizosphere microbiome functions

**Figure 5:**
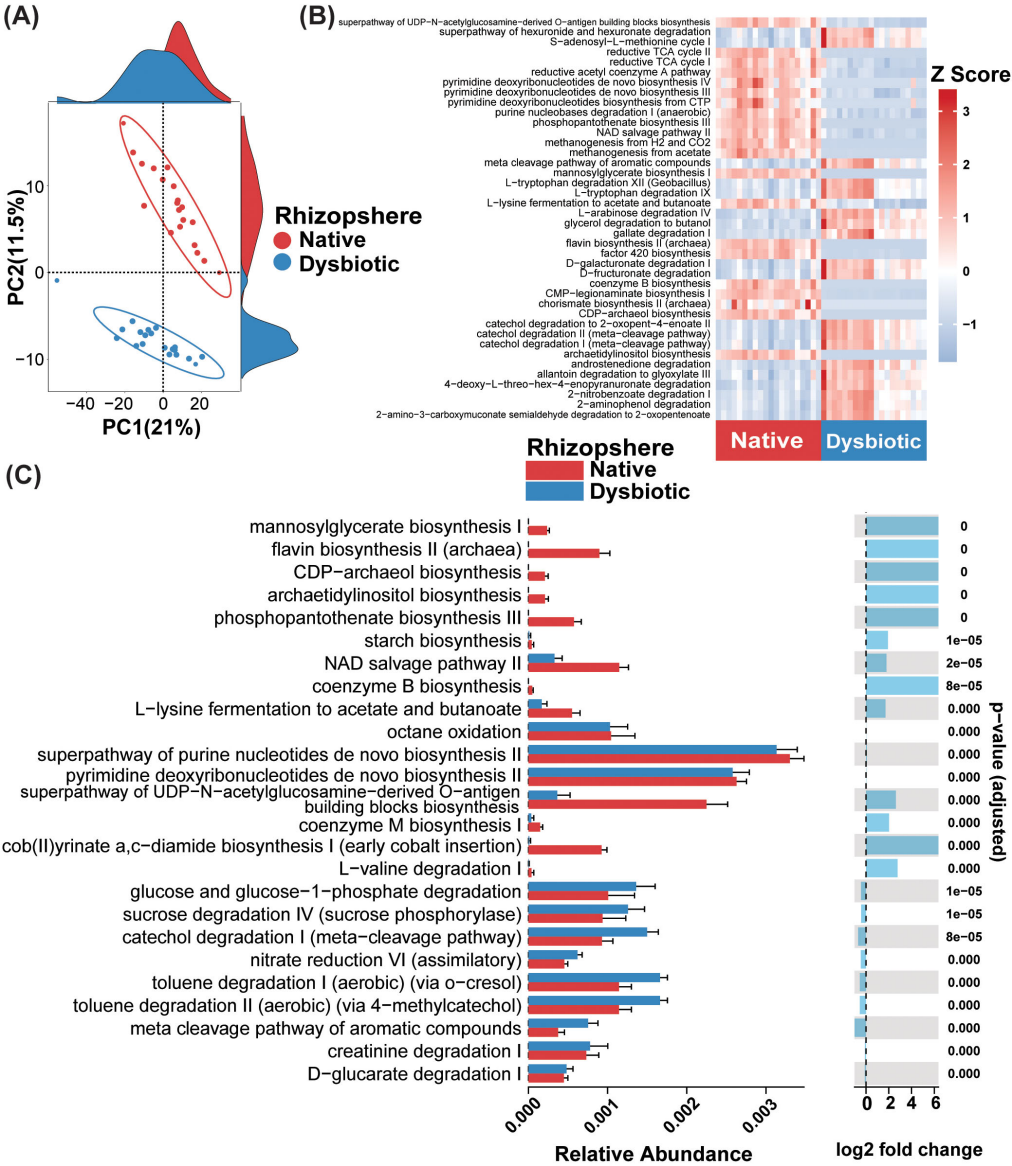
PICRUSt2-predicted metabolic pathway profiles of cucumber rhizosphere prokaryotic communities under native and dysbiotic soil conditions. **(A)** Principal component analysis (PCA) shows distinct separation in metabolic pathway profiles, highlighting functional differences between native and dysbiotic rhizosphere microbial communities. **(B)** Heatmap illustrating significant variations in the predicted metabolic pathway abundances between native and dysbiotic soil conditions. **(C)** Bar plots representing the relative abundances and log2 fold-changes of the 25 most abundant metabolic pathways. Biosynthetic pathways contributing to structural integrity and microbial community stability were predominantly enriched in the native rhizosphere, whereas pathways associated with plant-microbe interactions such as glucose and glucose-1-phosphate degradation and sucrose degradation IV showed enrichment in the dysbiotic rhizosphere.

A clear divergence between the predicted metabolic pathways in the bacterial communities of the native and dysbiotic rhizospheres was observed (**Figure 5A**). Moreover, a significant deviation in pathway abundance from the mean was noted in the metabolic pathway heatmap, indicating functional shifts in the assembled rhizosphere microbiome under dysbiotic conditions (**Figure 5B**). Pathways that were significantly enriched in the native rhizosphere tended to be depleted in the dysbiotic rhizosphere, and vice versa, suggesting an inverse relationship in metabolic activity in the two rhizospheres. Additionally, analysis of metabolic pathway abundance and its log2-fold change revealed highly significant differences between native and dysbiotic rhizospheres (**Figure 5C**). Metabolic pathways related to sugar metabolism, including glucose and glucose-1-phosphate degradation and sucrose degradation IV (sucrose phosphorylase), as well as those related to nitrogen and cofactor metabolism, including nitrate reduction VI (assimilatory) and creatinine degradation I, were significantly more abundant (p < 0.001; **Figure 5C**) in the dysbiotic rhizosphere than in the native rhizosphere. Metabolic pathways involved in aromatic compound degradation, such as catechol degradation I and toluene degradation I and II, were also enriched in the dysbiotic rhizosphere. In contrast, pathways associated with microbial cell wall and signal molecule biosynthesis, including the superpathway of UDP-N-acetylglucosamine-derived O-antigen building block biosynthesis and phosphopantothenate biosynthesis III (CoA biosynthesis), were significantly enriched in the native rhizosphere compared with the dysbiotic rhizosphere. Similarly, pathways related to amino acid and vitamin biosynthesis, such as L-lysine fermentation to acetate and butanoate, flavin biosynthesis II, cob(II)yrinate a, and c-diamide biosynthesis I (vitamin B12 biosynthesis), were significantly enriched in the native rhizosphere but depleted in the dysbiotic rhizosphere (p < 0.001; **Figure 5C**).

In the dysbiotic rhizosphere, bioagent application resulted in metabolic pathways that were clearly distinct from metabolic pathways in the untreated control; however, in the native rhizosphere, the metabolic profiles remained less distinct from metabolic profiles in the untreated control (**Supplementary File: Supplementary Figure S4A, S4B**).

### Transcriptomic analysis of *Cucumis sativus* L. reveals bioagent integration under dysbiotic conditions

**Figure 6:**
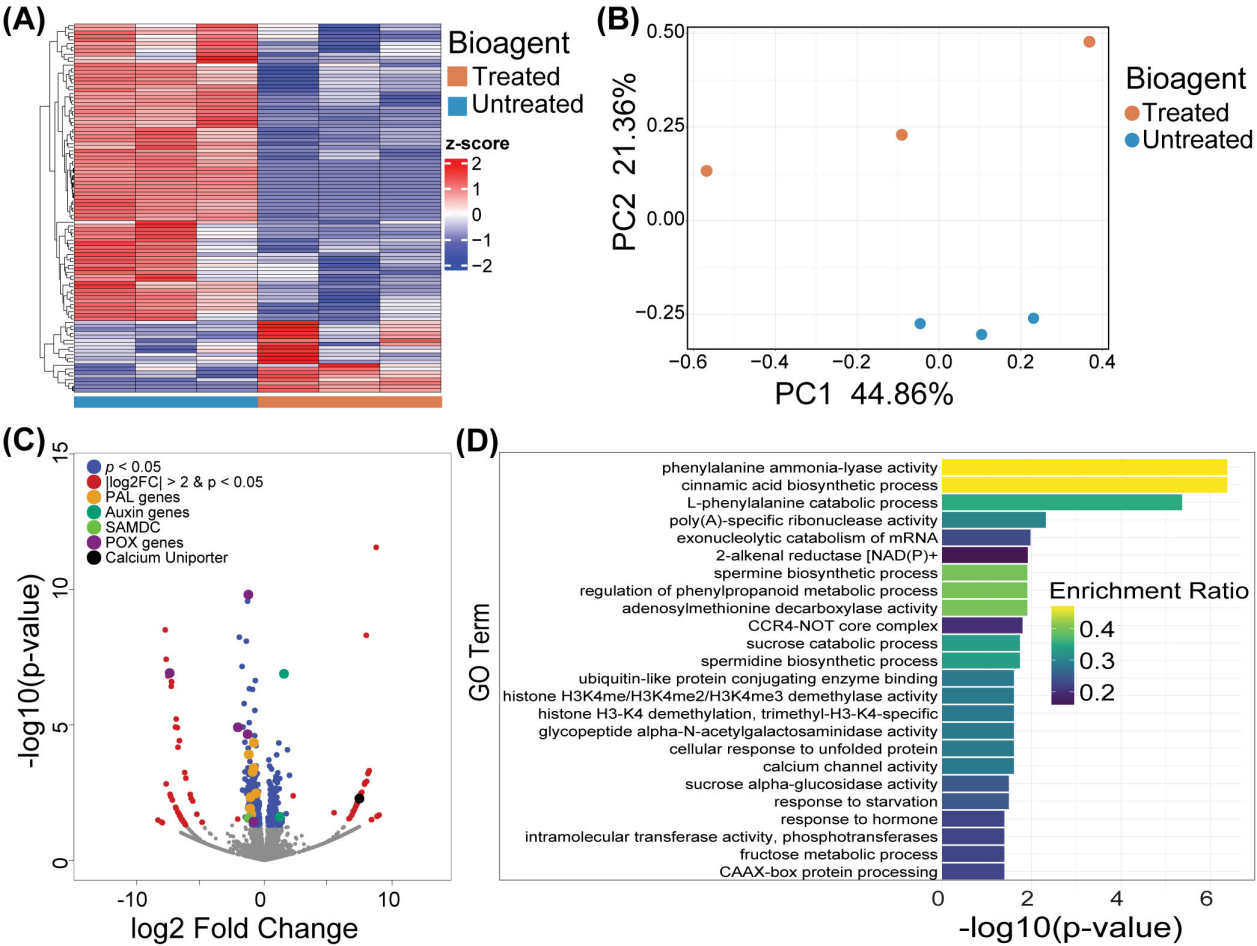
Transcriptomic responses of cucumber seedlings to bioagent inoculation in dysbiotic soils. **(A)** Hierarchical clustering heatmap of differentially expressed genes (DEGs) delineates distinct transcriptional profiles between bioagent-inoculated and untreated (control) *Cucumis sativus* seedlings, reflecting divergent gene expression patterns. **(B)** Principal Component Analysis (PCA) ordination exhibits clear separation between bioagent-treated and untreated groups, underscoring a robust treatment effect on the seedling transcriptome. **(C)** Volcano plot of DEGs highlights significant transcriptional changes, with downregulation of defense-related genes (PAL, POX, SAMDC) and upregulation of genes associated with auxin signaling and calcium transport, suggesting defense priming coupled with energy reallocation from defense to growth processes. **(D)** Gene Ontology (GO) enrichment analysis of DEGs reveals overrepresented biological processes, indicating activation of broad-spectrum defense mechanisms and efficient defense priming under cost-effective conditions.

Quality filtering removed 1,355,029 read pairs (3.49%), retaining 34,386,784 clean paired-end reads (88.66%), along with 5.07% forward-only and 2.78% reverse-only reads. The clean reads were aligned to the *Cucumis sativus* reference genome (GCF_000004075.3_Cucumber_9930_V3) (Huang et al., 2009) using HISAT2 (Kim et al., 2015), achieving an overall alignment rate of 89.27% (Supplementary file: **Supplementary Table S10**). Transcriptomic profiling of *Cucumis sativus* L. revealed that bioagent inoculation effectively integrates with the plant’s regulatory networks, activating metabolic pathways critical for defense and growth. Hierarchical clustering of differentially expressed genes (DEGs), visualized via heatmap analysis (**Figure 6A**), revealed distinct transcriptional signatures between bioagent-inoculated and uninoculated seedlings. Principal component analysis (PCA) confirmed the clear separation of the gene expression profiles between the treated and untreated groups (**Figure 6B**). Volcano plot analysis further revealed significant DEGs in bioagent-treated plants, with notable downregulation of phenylalanine ammonia-lyase (PAL) genes (CsaV3_6G039690, CsaV3_4G002320, CsaV3_4G002310, CsaV3_4G002290), peroxidase (POX) genes (CsaV3_3G042040, CsaV3_7G006370, CsaV3_7G005720, CsaV3_3G035690), and the defense-associated S-Adenosylmethionine Decarboxylase gene (SAMDC gene) (CsaV3_2G007750) (**Figure 6C**). Conversely, the upregulation of auxin-related genes (CsaV3_2G01320, CsaV3_1G009120) and the calcium uniporter gene (CsaV3_6G042480) has been detected (**Supplementary File: Supplementary Table S12**). The bioagent-driven response favored growth-associated processes, with upregulation of calcium uniporter genes facilitating cytosolic calcium sequestration to avoid toxicity while sustaining calcium-mediated signaling for symbiotic colonization and pathogen recognition (**Figure 6C**). Gene Ontology (GO) enrichment analysis also revealed significant enrichment of biological processes, including cinnamic acid biosynthesis, phenylalanine ammonia-lyase activity, and the regulation of phenylpropanoid metabolism, which are essential for the production of antimicrobial phenolics, lignin, and polyphenols that bolster plant immunity (**Figure 6D**). Bioagent inoculation in cucumber led to upregulated calcium channel activity, highlighting the role of Ca^2+^signaling in early immune activation. Concurrently, hormone-responsive genes were induced, indicating coordinated regulation of defense responses (**Figure 6D**). Hence, bioagent inoculation significantly altered cucumber gene expression profiles, promoting a balance between growth and immune responses in dysbiotic soils.

### Phenotypic responses reflect rhizosphere microbiome and transcriptomic alterations

**Figure 7:**
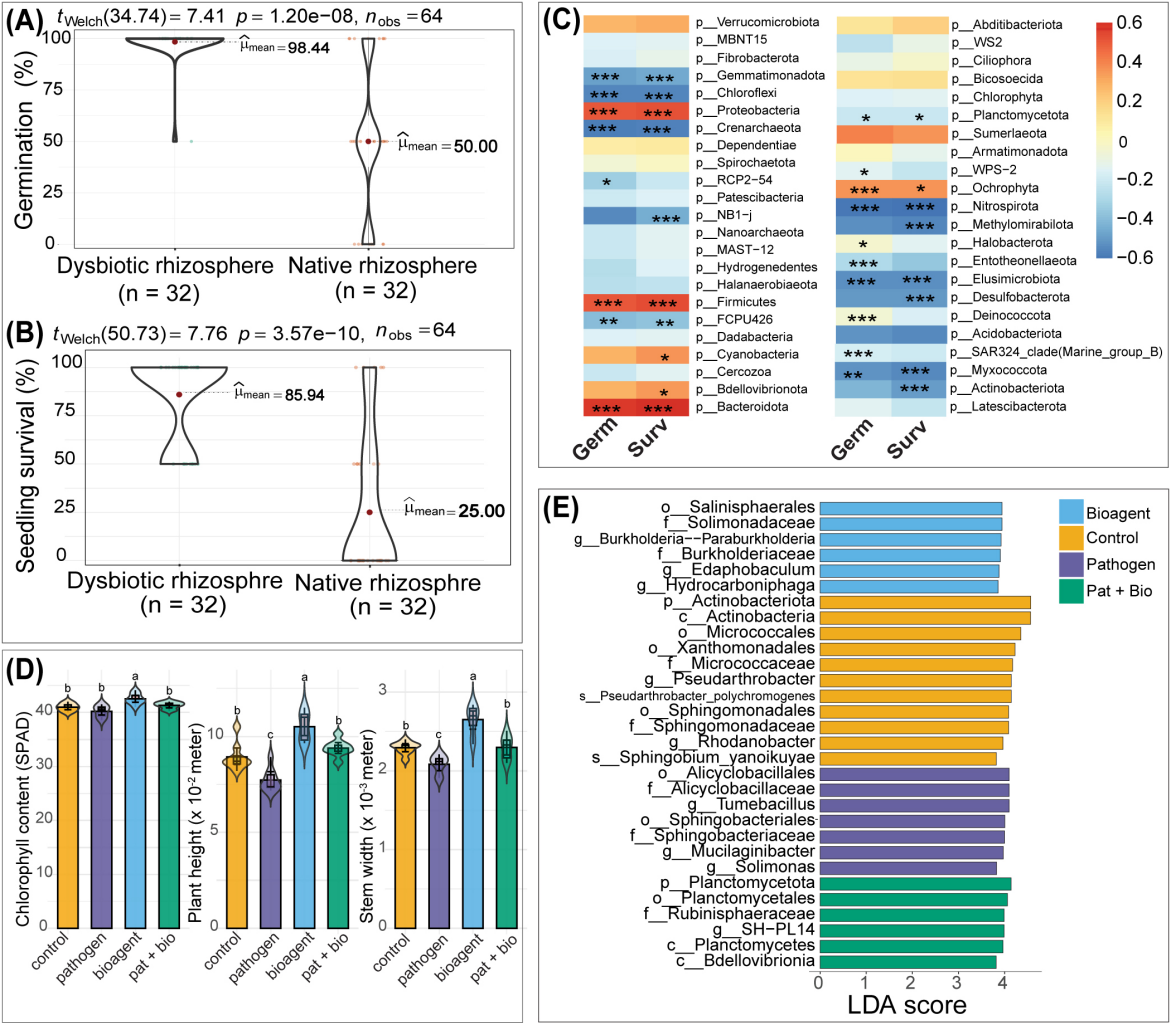
Phenotypic responses of *Cucumis sativus* seedlings to MHT and microbial inoculations. **(A, B)** Welch’s t-test demonstrates significantly higher seed germination **(A)** and seedling survival rates **(B)** in the dysbiotic rhizosphere than the native rhizosphere. **(C)** Correlation analysis illustrating associations between prokaryotic phylum abundances and seed germination and seedling survival rates. *, **, and *** denote significant correlations at *p* < 0.05, 0.01, and 0.001, respectively. (*p* < 0.001). **(D)** ANOVA followed by Tukey’s HSD multiple comparisons indicates superior growth responses in seedlings inoculated with bioagents within the dysbiotic rhizosphere. Groups sharing identical letters are not significantly different at *p* < 0.05.” **(E)** Differential abundance of core microbiome taxa following microbial inoculations in the dysbiotic rhizosphere showing differential enrichment of common plant growth promoting bacteria under bioagent inoculation.

Phenotypic responses in *Cucumis sativus* L. seedlings under dysbiotic conditions corresponded with rhizosphere microbiome shifts in the dysbiotic rhizosphere and transcriptomic reprogramming induced by bioagent inoculation. Seed germination rates were significantly higher in the dysbiotic rhizosphere than in the native rhizosphere (**Figure 7A**), with seedling survival rates markedly elevated in the dysbiotic rhizosphere (85.9% vs. 25%; p < 0.001; **Figure 7B**). Seedling germination and survival rates in the native and dysbiotic rhizospheres were positively correlated with increased abundances of Bacteroidota, Firmicutes, and Proteobacteria, and negatively correlated with Chloroflexi, Crenarchaeota, Nitrospirota, and Myxococcota (**Figure 7C**).

In the dysbiotic rhizosphere, seedling survival among the microbial treatments and the control was not statistically significant (Kruskal–Wallis, p = 0.155; **Supplementary File: Supplementary Figure S5A**). However, bioagent inoculation significantly improved growth parameters, including stem diameter (p < 0.01), chlorophyll content (p < 0.01), and plant height (p < 0.05; **Figure 7D**). In the native rhizosphere, seedling survival, although low, did not differ significantly among microbial treatments (Kruskal–Wallis, p = 0.155; **Supplementary File: Supplementary Figure S5B**). As 75% of seedlings had died by day 30 in the native rhizosphere, phenotypic data were insufficient for robust statistical comparisons in the native rhizosphere.

Kruskal–Wallis tests assessing overall differences in taxonomic enrichment across microbial inoculation groups indicated that no taxa were significantly enriched among treatments in the native rhizosphere (p > 0.05). In contrast, the dysbiotic rhizosphere exhibited significant enrichment of specific taxa across treatments (p < 0.05). Linear discriminant analysis (LDA) further identified taxa contributing to significant differences among microbial inoculation in the dysbiotic rhizosphere, with bioagent treatment favoring beneficial taxa such as Paraburkholderia and members of the Burkholderiaceae family (**Figure 7E**). Redundancy analysis (RDA) further confirmed the association of these beneficial taxa with bioagent treatment under dysbiotic rhizosphere conditions (**Supplementary File: Supplementary Figure S6**).

## Discussion

The resident soil microbiota harbors complex interactions among microbial communities, contributing to its resilience against biotic and abiotic disturbances (Lourenço et al., 2018a; Zhikang et al., 2021b). Thus, a microbial inoculum with demonstrated growth-promoting or pathogen-antagonistic properties under controlled conditions may fail to achieve the intended effects in natural environments, where complex microbial interactions prevail. Although several studies have highlighted the limited ability of microbial inoculants to establish in the rhizosphere or deliver their intended benefits (Thomas and Sekhar, 2016; Zhikang et al., 2021a; Rasool et al., 2025a) the mechanisms by which resident soil microbiota influence inoculation outcomes and plant health remain insufficiently elucidated. To address this, we subjected the soil to MHT which induced a state of dysbiosis characterized by significant alterations in relative abundance, QA and predicted functions of the native microbial community. These changes indicate substantial functional shifts that could be linked to microbial interactions and overall ecosystem functioning. In particular, MHT-induced dysbiosis generated a microbial gradient that could reshape both plant–microbe and microbe–microbe interactions in the soil (Jing et al., 2022), thereby providing a useful platform to investigate the role of resident soil microbiota in inoculation success and plant health. Importantly, MHT caused insignificant alterations in soil chemical properties, underscoring its reliability for studying microbiome dynamics without confounding effects from altered nutrient profiles (Beugnon et al., 2021). Moreover, MHT offers an advantage over antibiotic-based approaches for inducing dysbiosis, as it avoids residual effects that might otherwise produce prolonged and unintended impacts on soil microbial communities (Ketehouli et al., 2024).

Quantitative microbiome profiling revealed a significant shift in absolute bacterial abundance, with depletion levels reaching 96.4% after MHT. However, cucumber planting facilitated the recovery of 78% of the lost microbial abundance, contributing to the enrichment of a healthier rhizosphere and fostering beneficial microbial activities for supporting plant health and growth. On the other hand, the unaltered rhizosphere maintained a strong legacy effect on the native microbial community, enriching the less healthy rhizosphere and resisting changes induced by microbial inoculation; which goes in line with the findings of Zhikang et al (2021a). The inability of microbial inoculants to establish in the rhizosphere may result from competitive interactions with the resident soil microbiota, which can increase the proliferation of indigenous microbial communities (Čaušević et al., 2024). The resident microbiome may also diminish the ability of a bioagent to induce the desired change, as demonstrated by a recent study showing that the resident soil microbiome reduces the capacity of *Metarhizium brunneum* to confer resistance to spider mites (Rasool et al., 2025a).

Although cucumber plants exhibited strong rehabilitative ability, recovering 78% of the lost microbial abundance and reestablishing a healthy rhizosphere after MHT, they could not surpass the inherent resilience of the native microbial community, as postplanting diversity remained nested within preplanting communities (**Figure 1G**). Given that biotic competition from the native microbiota is often overlooked (Albright et al., 2022), microbial inoculant–driven restructuring of the rhizosphere may fail to achieve the desired outcomes (Thomas and Sekhar, 2016). Thus, inducing a temporary dysbiosis of the resident soil microbiota may increase compatibility with inoculants and facilitate the intended community shifts (Kaminsky et al., 2019).

The regression models indicated that higher QA was associated with a reduced germination percentage and seedling survival. Specifically, each unit increase in standardized QA corresponds to a 0.457% decrease in germination percentage and a 0.452% decrease in seedling survival. The R² values suggest that QA explained 45.8% of the variance in germination percentage and 36.1% of the variance in seedling survival, indicating moderate to strong predictive power. These negative correlations align with the observed negative correlations (r = -0.68 for germination, r = -0.60 for survival), reinforcing that higher microbial abundance may suppress *C. sativus* performance, potentially due to increased microbial competition or pathogen load in the rhizosphere (Guo et al., 2020; Yan et al., 2022). This finding is consistent with the SEM results, where QA mediated the effects of MHT on microbial diversity and plant outcomes, with reduced QA linked to increased germination and survival via altered diversity metrics as demonstrated in **Figure 2E**.

Microbial network complexities have also provided good insights into the resilience of microbial communities under biotic and abiotic stresses (Hernandez et al., 2021; Gao et al., 2022; Pechlivanis et al., 2024). Microbial abundance, which contributes to network complexity, has been associated with the resistance of a native microbiome to invasions by exotic communities (Vivant et al., 2013; Mallon et al., 2015). Thus, the resilience of the resident rhizosphere microbial community arises from its highly diverse and interconnected network, where functional coordination among taxa establishes a robust ecological barrier, mitigating the impact of external microbial introductions. This network helps the microbiome resist the effects of microbial inoculation and perturbs plant-derived hormonal and nutrient signals released through root exudates (Li et al., 2024a). Since cucumber assembles its rhizosphere community primarily by recruiting microbes from the soil microbiota, the recruited microbes from the native soil could retain the inhibitory legacy of the resident microbiome, limiting the ability of introduced inoculants to restructure the rhizosphere unless this legacy is disrupted through MHT.

The enrichment of predicted metabolic pathways associated with microbial cell wall biosynthesis, signal molecule production, and amino acid and vitamin biosynthesis in the native rhizosphere suggests that the microbial community maintains structural integrity and metabolic self-sufficiency, potentially enhancing resistance to external disturbances. These pathways likely contribute to reinforcing microbial resilience by supporting cellular stability, energy metabolism, and the synthesis of secondary metabolites involved in microbial communication and defense (Barreteau et al., 2008; Li et al., 2024b). In contrast, the enrichment of pathways related to sugar metabolism, nitrogen and cofactor metabolism, and aromatic compound degradation in the dysbiotic rhizosphere suggested functional reconfiguration toward plant-associated interactions. This shift implies increased microbial reliance on plant-derived carbon sources and increased metabolic cooperation in nutrient cycling (Oyserman et al., 2022; Chen et al., 2023b). This also indicates a potential role in plant defense by facilitating the degradation of aromatic compounds, which may include plant-derived secondary metabolites with antimicrobial properties. In the rhizosphere, microbes capable of degrading these compounds may adapt to plant-derived exudates and could contribute to modulating microbial interactions, including pathogen suppression (Rogowska-van der Molen et al., 2023).

The phenotypic responses of cucumber seedlings to MHT and microbial inoculation substantiate the predicted functional roles of rhizosphere microbial communities. The quantitative microbial abundance has emerged as a key determinant of these phenotypic responses, affecting germination and seedling survival. In the native rhizosphere, which is resilient to environmental perturbations, a relatively high total microbial abundance negatively affects plant health by constraining the ability of a plant to effectively modulate its rhizosphere microbiome. Consequently, the introduction of bioagents, which could facilitate microbiome reorganization in the dysbiotic rhizosphere, failed to achieve similar restructuring in the native rhizosphere. This could be attributed to the bioagent-induced increase in total microbial abundance, which was three times greater in the native rhizosphere than in the dysbiotic rhizosphere. This increased microbial load likely increased microbial network complexity, thereby increasing the resistance of the native rhizosphere to restructuring (Gao et al., 2022; Pechlivanis et al., 2024). Pathogen inoculation in the native rhizosphere significantly reduced the total microbial abundance, resulting in minimal impacts on microbial network complexity. This might have resulted in seedling survival rates comparable to those in the untreated control. This effect may be associated with the activation of plant immunity via the “cry-for-help” strategy (Liu et al., 2021b; Yin et al., 2021), which likely mitigates the presence of various rhizosphere pathogens.

From a soil degradation perspective, greater microbial diversity is widely regarded as beneficial for maintaining soil health, as it provides functional redundancy that enhances the system’s resilience to disturbances (Chen et al., 2020; Coban et al., 2025). However, it is increasingly evident that microbial diversity alone does not necessarily predict plant health outcomes. Variations in microbial load, such as those we observed between dysbiotic and native rhizospheres, can substantially affect plant performance. While relative abundance metrics are often used to infer microbial community impacts on host phenotypes, they overlook the influence of total microbial biomass, potentially masking key drivers of plant health (Vandeputte et al., 2017). In this context, quantitative microbiome profiling offers a more robust framework for explaining the observed phenotypic responses in *Cucumis sativus* L. seedlings under dysbiotic conditions by integrating both QA and community composition into the analysis.

Bioagent-treated seedlings demonstrate improved growth through both direct physiological effects and indirect benefits mediated by a restructured rhizosphere microbiome. The bioagent selectively enriches beneficial microbial taxa such as Paraburkholderia and members of Burkholderiaceae, promoting functionally supportive community even under dysbiotic soil conditions (Toju et al., 2018). In contrast, the native rhizospheres lacked consistent taxonomic enrichment across treatments. These results underscore that plant performance in dysbiotic soils depends more on the functional enhancement of specific taxa than on total diversity, positioning bioagent inoculation as a promising tool for restoring soil health and improving crop productivity (Finkel et al., 2020).

Transcriptomic profiling revealed that bioagent inoculation acts as a form of immune priming, akin to mild vaccination, inducing systemic resistance without activating the energetically costly responses typically associated with pathogenic attack (Pieterse et al., 2014).

**Figure 8:**
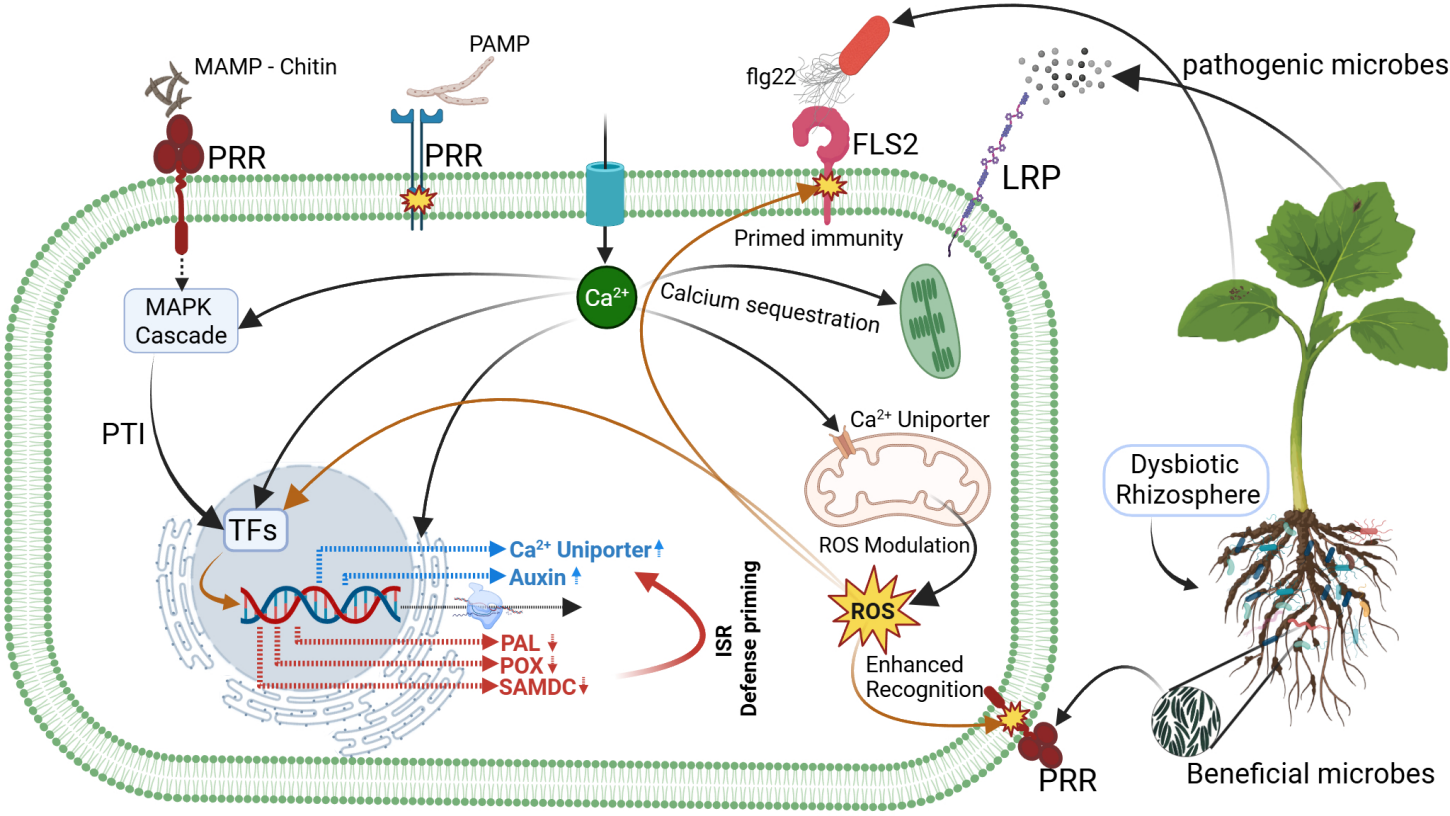
Schematic representation of defense priming in cucumber cells. Perception of chitin from bioagent inoculation by cell-surface PRRs activates MAPK signaling, calcium influx, and pattern-triggered immunity (PTI) responses. Because the signal derived from a beneficial source, excess cytosolic calcium is sequestered into intracellular sinks and mitochondria, resulting in ROS modulation and transcriptional downregulation of defense-related genes (e.g., *PAL*, *POX*, *SAMDC*), while auxin-associated genes are upregulated to support growth. In parallel, calcium signaling maintains PRRs in a primed state, enhancing the plant’s capacity for rapid recognition of both mutualistic and pathogenic microbes.

Upon recognition of chitin as a microbe-associated molecular pattern (MAMP) derived from the bioagent, cell surface pattern recognition receptors (PRRs) initiate a signaling cascade through Mitogen-Activated Protein Kinases (MAPKs). This triggers calcium influx, contributing to the activation of pattern-triggered immunity (PTI) and initiating basal defense responses, revealed through significant enrichment of calcium channel activity (**Figure 6D**). However, as the chitin signal originated from a non-pathogenic (beneficial) source, the plant modulates its response by sequestering excess cytosolic calcium into calcium sinks and mitochondria, a process reflected by the upregulation of calcium uniporter genes (Galotto et al., 2020; **Figure 8**). This regulated calcium homeostasis modulates reactive oxygen species (ROS) levels, as calcium signaling and ROS are intricately linked in plant defense signaling which in turn modulates transcriptional responses (Yuan et al., 2022). This resulted in downregulation of defense-related genes such as PAL, POX, and SAMDC which minimizes unnecessary energy expenditure on defense and supports a metabolic shift toward growth, as evidenced by the activation of auxin-related genes (Yu, et al., 2022; Ma et al, 2022; **Figure 6C**). Simultaneously, ROS modulation helps maintain cell-surface receptors in a primed state, thereby enhancing recognition of both mutualistic microbes and potential pathogens (e.g. via the flg22–FLS2 axis). This balance ensures energy-efficient growth while preserving immune sensitivity (Kadota et al., 2015; **Figure 8**).

This primed state was maintained for at least 30 days post-inoculation in our study, which is consistent with prior findings showing that induced systemic resistance (ISR) can persist for several weeks and, in some cases, even extend across plant generations through epigenetic mechanisms (Slaughter et al., 2012; Cooper and Ton, 2022). The ISR is known to involve a coordinated network of metabolic adjustments, immune pathway activation, and stress modulation, including the regulated expression of pathogenesis-related (PR) proteins, redox balance, and hormone signaling pathways (Pieterse et al., 2014; Romera et al., 2019). Our transcriptomic analysis mirrored this coordination, with enriched GO terms related to phenylpropanoid metabolism, cinnamic acid biosynthesis, and hormone-mediated signaling, supporting the activation of broad-spectrum defense pathways (**Figure 6D**). Interestingly, while these defense-related GO terms were upregulated, core structural defense genes such as those in the PAL and POX families were downregulated.

This pattern aligns with the ISR strategy of maintaining plants in a low-cost, primed state, enabling faster and stronger responses upon subsequent pathogen exposure without constitutive activation (Wilkinson et al., 2019). Concurrently, the upregulation of auxin-responsive genes and calcium signaling components suggests that bioagent inoculation also promotes growth-supporting processes and prepares the plant for beneficial microbe recognition and balanced immune activation (Mauch-Mani et al., 2017).

## Conclusions

Modification of the rhizosphere community by strategically modulating resident soil microbial load before planting, combined with targeted bioagent inoculation, offers a promising framework for achieving effective and directed reorganization of the rhizosphere microbiome by boosting bioinoculant efficacy. Our findings demonstrate that phenotypic resilience in cucumber under dysbiotic conditions emerges not only from changes in microbial community composition but also from modulated microbial load which triggered host transcriptomic reprogramming. Crucially, these insights highlight the limitations of relying solely on relative abundance metrics to interpret plant–microbiome interactions, underscoring the added explanatory power of quantitative microbiome profiling, which captures shifts in microbial load alongside community restructuring. By leveraging the preexisting soil microbial pool and priming it to accommodate beneficial inoculants, this two-step strategy may overcome the resistance to changes often observed in resilient pathogenic soils, where high microbial load and complex networks can buffer against external interventions. Properly calibrated, such an approach could enable bioinoculants to establish more effectively, creating a restructured rhizosphere that promotes plant health without inducing the unintended consequences of microbial overload. Moving forward, our research will explore more specific calibrations and investigate the fungal dimension of these interactions, recognizing the pivotal roles fungi play in nutrient cycling, soil health, and plant–microbe symbioses.

## Data Availability

The raw sequences of 16S rRNA amplicon sequencing of pre-planting soil microbiome, post-planting rhizosphere microbiome, and the raw RNA-Seq reads generated from *Cucumis sativus* seedlings have been deposited in DDBJ/ENA/GenBank and can be accessed at https://www.ncbi.nlm.nih.gov/bioproject/PRJNA1234063 under BioProject PRJNA1234063.
