# Supplementary material for: Soil microbial load modulation improves plant–microbe interactions and bioinoculant efficacy in pathogen-stressed soils
Source: Front Plant Sci. 2025 Dec 4;16:1712997. doi: 10.3389/fpls.2025.1712997 (PMC12711777; doi:10.3389/fpls.2025.1712997)
Supplement: Supplementary file 1 [file DataSheet1.docx]

**Supplementary file**

**Article title**: **Soil microbial load modulation improves plant–microbe interactions and bioinoculant efficacy in pathogen-stressed soils**

Yohannes Ebabuye Andargie ^1,2^, GyuDae Lee^3^, Min-Ji Kim^4^, Eskindir Getachew Fentie ^1^, Minsoo Jeong^1^, Setu Bazie Tagele^5^, Kyeongmo Lim^1^, Ugur Azizoglu^6,7^, Jae-Ho Shin^1,3,8*^

^1^NGS Core Facility, Kyungpook National University, Daegu, Republic of Korea; ^2^Department of Plant Sciences, Bahir Dar University, Bahir Dar, Ethiopia; ^3^ Department of Applied Biosciences, Kyungpook National University, Daegu, Republic of Korea; ^4^Department of Food Science and Nutrition, Pukyong National University, Busan 48513, Republic of Korea; ^5^Department of Microbiology and Plant Pathology, University of California Riverside, Riverside, CA 92507; ^6^Department of Crop and Animal Production, Safye Cikrikcioglu Vocational College, Kayseri University, Kayseri, Türkiye; ^7^Genome and Stem Cell Research Center, Erciyes University, Kayseri, Türkiye; ^8^Department of Integrative Biology, Kyungpook National University, Daegu, Republic of Korea

*Author for correspondence:

Jae-Ho Shin

**The following supplementary files are available for this article**


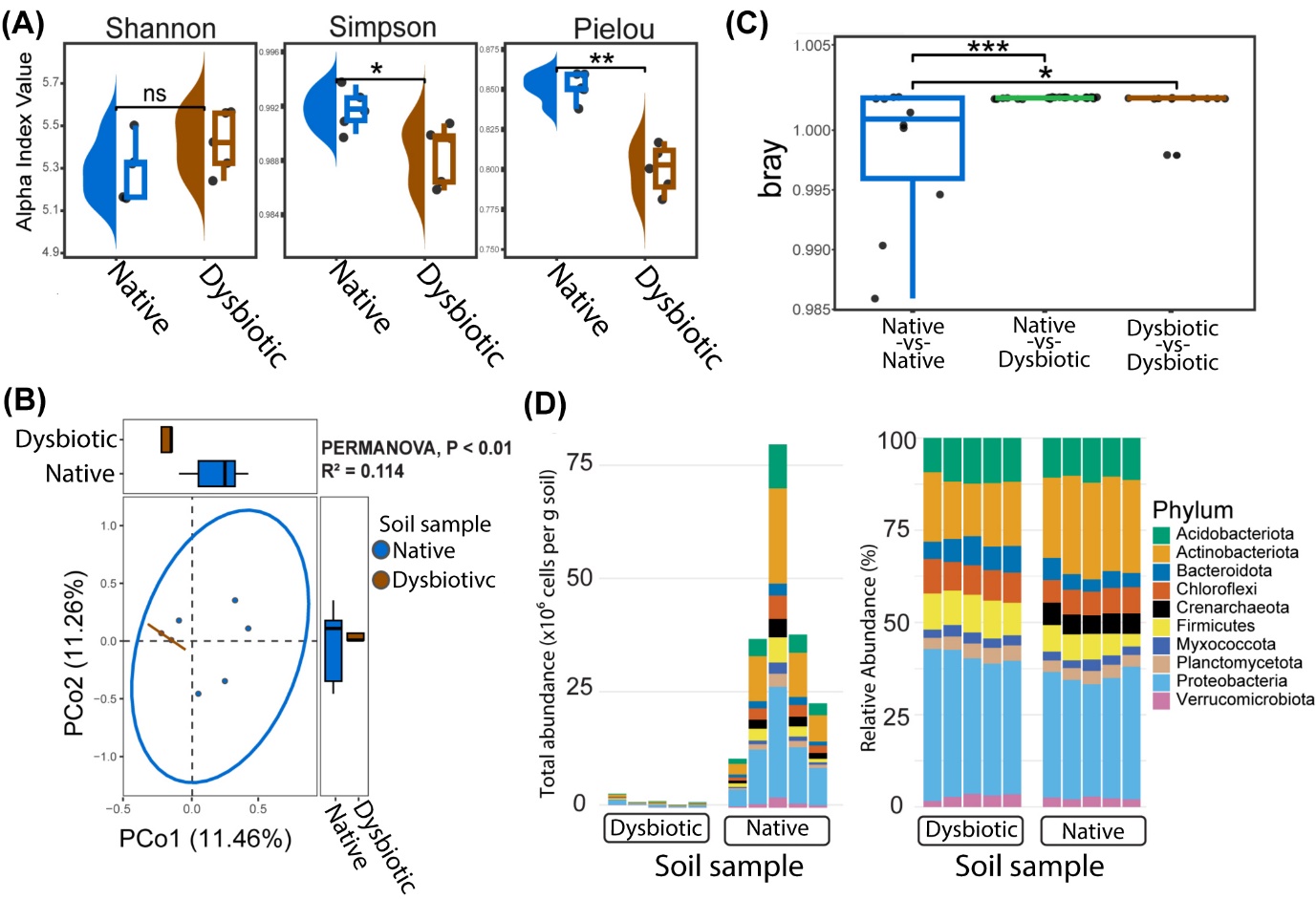
**Figure S1** Characterization of moist heat-induced dysbiosis in soil prokaryotic communities using relative and quantitative microbiome profiling. (A) Alpha diversity indices (Simpson and Pielou) indicate uneven bacterial diversity in heat-treated communities. (B) Principal coordinate analysis (PCoA) reveals significant differences in bacterial diversity dysbiotic and native soils. (C) Bray–Curtis dissimilarity highlights compositional shifts between dysbiotic and native soils. (D) Bar plots show the absolute and relative abundances of the top 10 resident prokaryotic phyla in in dysbiotic and native soils, highlighting an immense reduction in microbial abundance and compositional shifts induced by MHT.

**
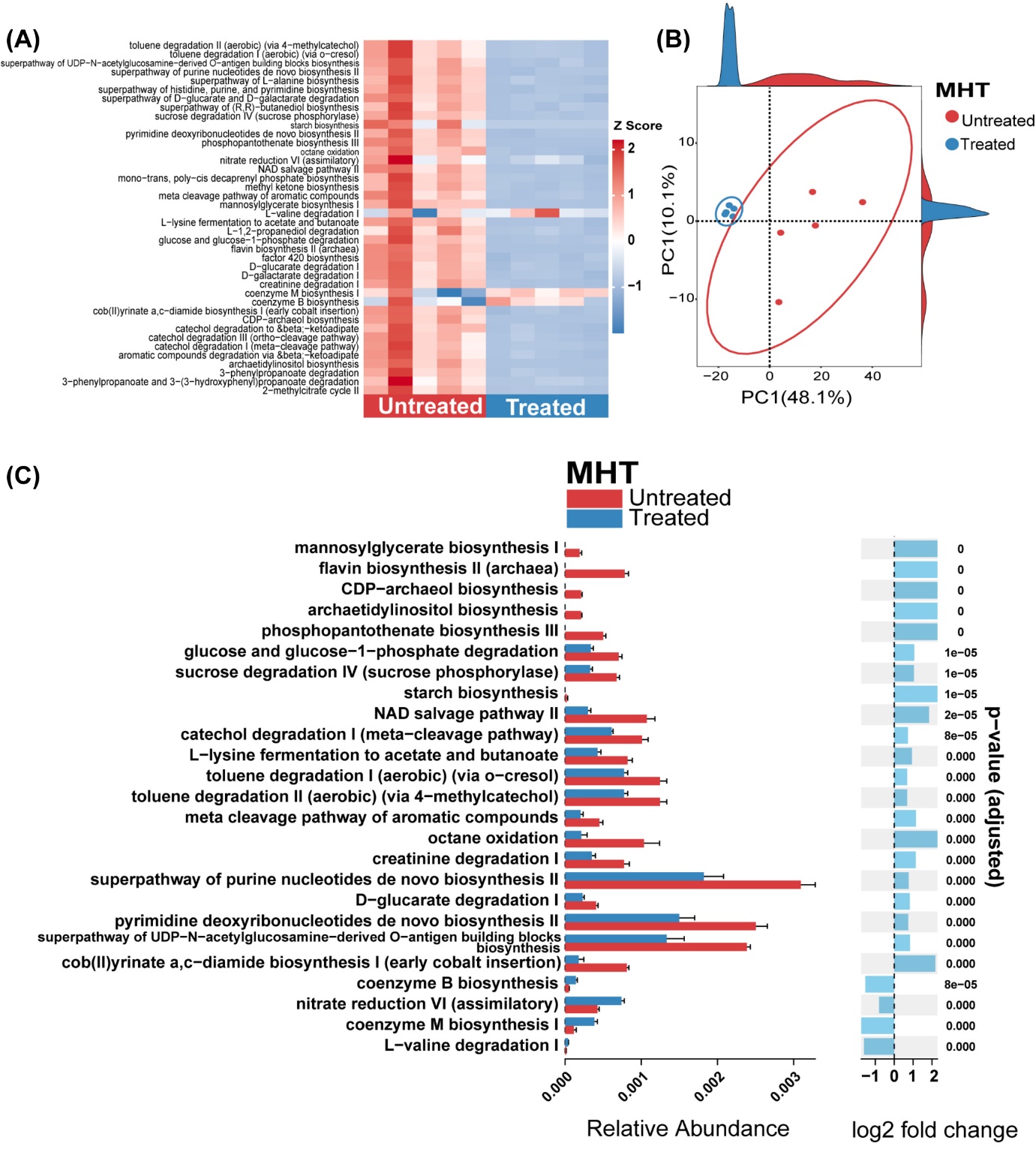
**

**Figure S2** Functional shift predictions in resident soil prokaryotes pre- and post-moist heat treatment before cucumber planting. (A) A metabolic pathway heatmap predicts functional alterations between treated (dysbiotic) and untreated (native) soil microbiomes. (B) Principal component analysis (PCA) illustrates distinct grouping of functional pathways between moist heat-treated (dysbiotic) and untreated (native) soil microbes. (C) Relative abundance and log2 fold change of the top 25 metabolic pathways highlight the microbial functional response to MHT.


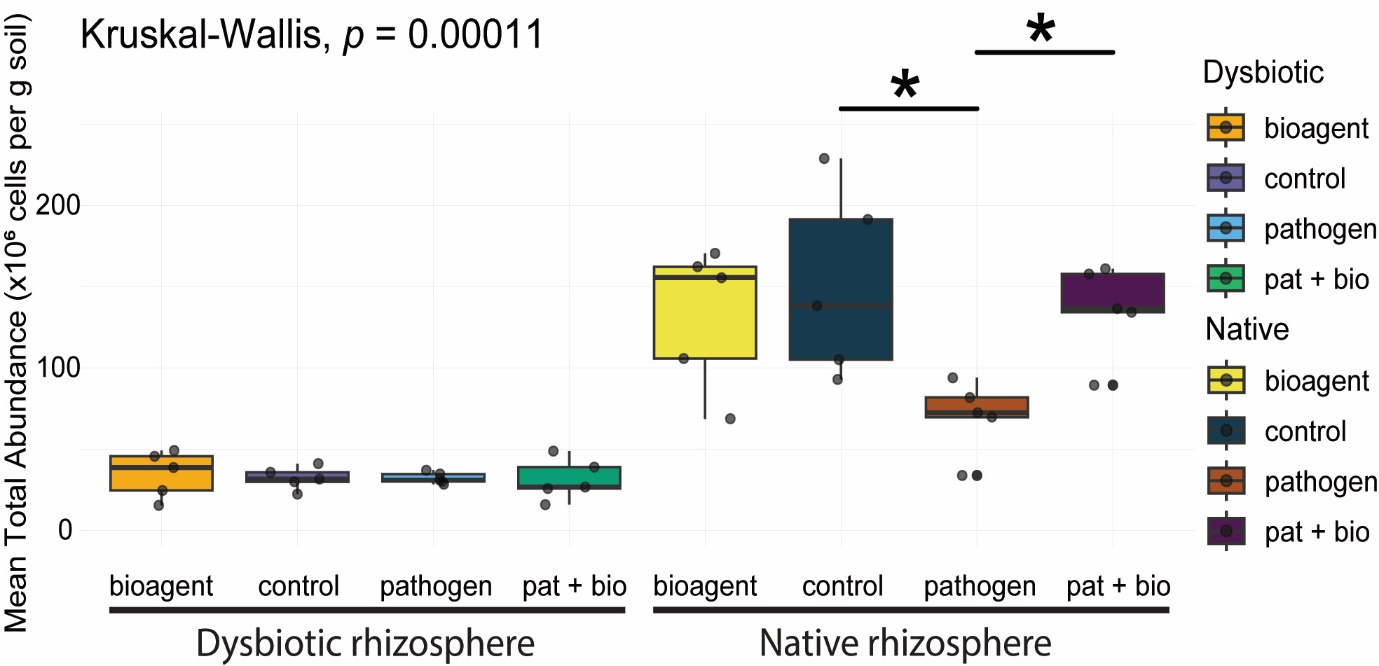


**Figure S3** Statistical Comparison of Bacterial Abundance in Native and Dysbiotic Rhizospheres. Kruskal-Wallis test showed significant bacterial abundance differences between dysbiotic and native bacteriomes. Pairwise Wilcoxon rank-sum test revealed lower abundance in pathogen-treated native rhizospheres, but no differences among microbial treatments in dysbiotic rhizospheres.


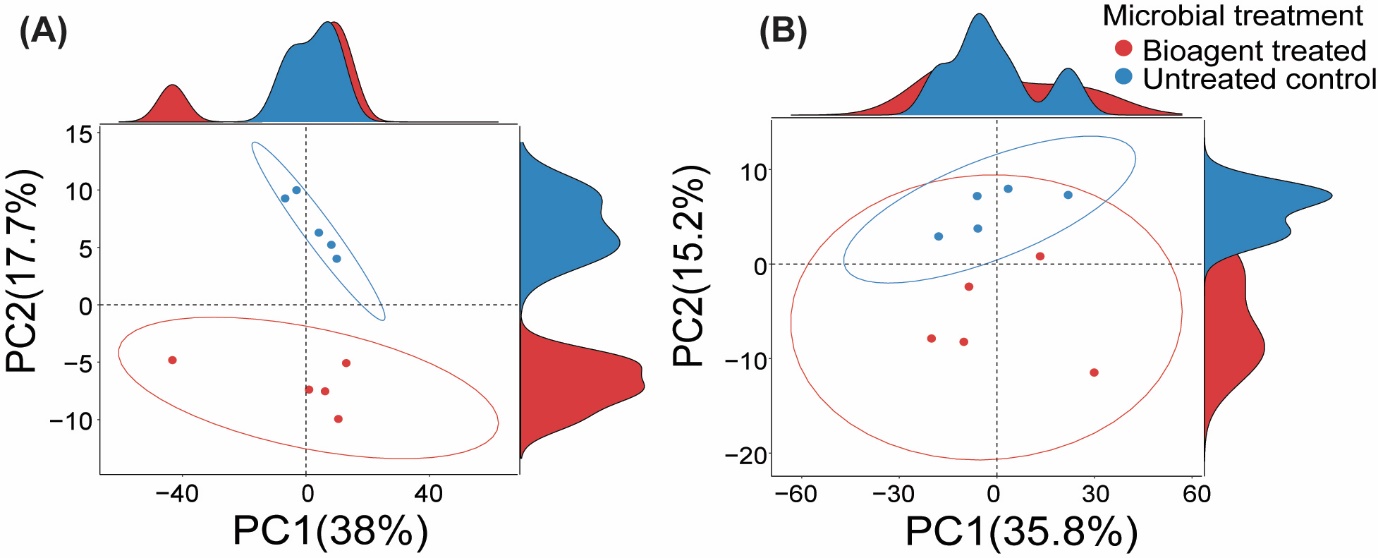


**Figure S4** Principal component analysis (PCA) of functional pathways under bioagent treatment. PCA illustrates distinct grouping of functional pathways between (A) bioagent treated and the untreated control in the dysbiotic rhizosphere and, (B) in the native rhizosphere.

**
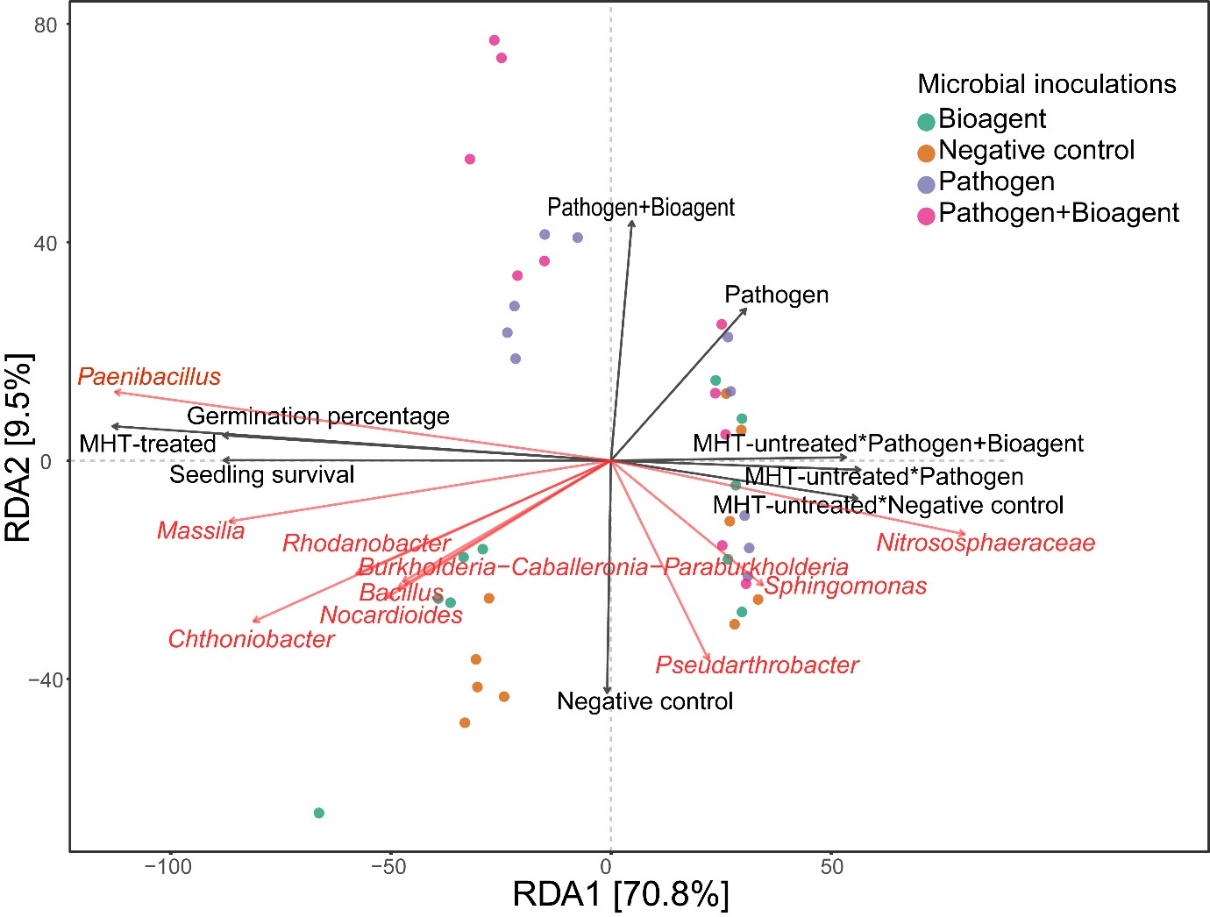
****Figure S5.** Kruskal–Wallis test showing no significant differences in cucumber seedling survival across microbial treatments (A) moist heat–treated (dysbiotic) rhizospheres and (B) untreated (native) rhizospheres, 30 days after planting.
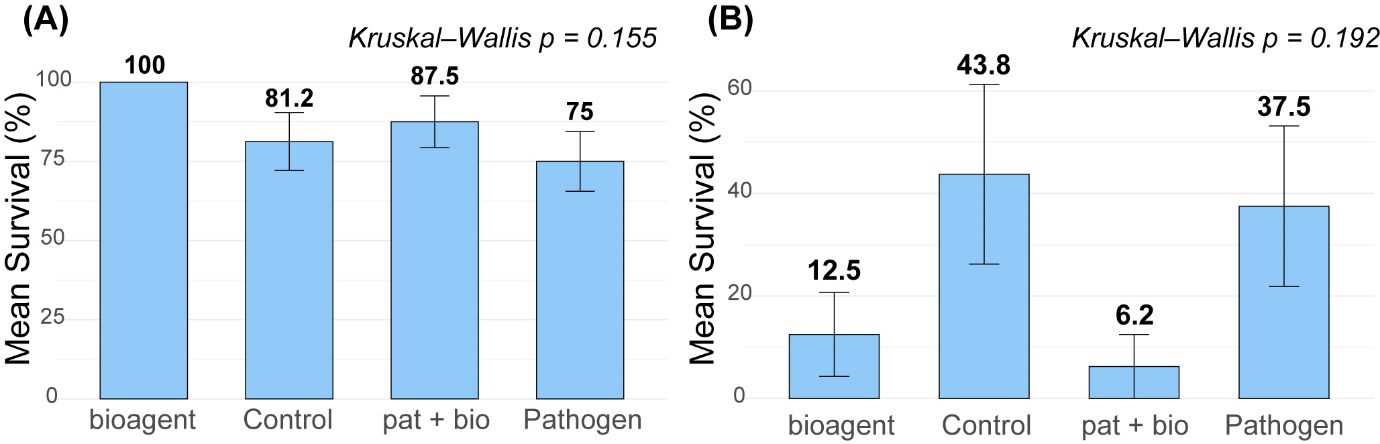


**Figure S6** Redundancy analysis showing relationships among of moist heat treatment, microbial inoculations, and bacterial genera. Bioagent treatment in the dysbiotic rhizosphere was closely associated with plant-related *Burkholderia* and *Bacilli* than in the native rhizosphere.

**Table S1** Physiochemical properties of moist heat-treated and untreated soils before cucumber planting.

| MHT | pH | TN  (%) | TOC  (%) | TP  (mg/Kg) | Ex-K  (cmolc/Kg) | Fe  (%) |
| --- | --- | --- | --- | --- | --- | --- |
| Untreated soil | 6.5 | 0.24 | 5.84 | 79.81 | 1.64 | 1.24 |
| Treated soil | 6.6 | 0.22 | 5.48 | 85.83 | 1.58 | 1.15 |

MHT = Moist heat treatment (at 105^o^C, 15 min), TN = Total nitrogen, TOC = Total organic carbon, TP = Total Phosphorus, Ex-K = Exchangeable potassium, Fe = Iron

**Table S2** Absolute abundance of prokaryotic communities in the resident soil and cucumber rhizosphere. The percentage of lost abundance after MHT and gained abundance after cucumber planting.

| MHT and cucumber planting | Mean Abundance  (x 10^6^ cells per g soil) | Standard Error | Mean Abundance (%) of untreated before planting | Standard  Error (%) |
| --- | --- | --- | --- | --- |
| Treated before planting | 1.43 | 0.37 | 3.6 | 0.9 |
| Untreated before planting | 40.1 | 12.4 | 100 | 30.9 |
| Treated after planting | 32.7 | 2.14 | 81.6 | 5.3 |
| Untreated after planting | 123 | 10.9 | 307 | 27.1 |

**Table S3**. Paired Wilcoxon signed-rank test of top 10 prokaryotic phyla between dysbiotic and native soils before planting.

| Phylum | .y. | Group 1 | Group 2 | n1 | n2 | Statistic | p | p-adj |
| --- | --- | --- | --- | --- | --- | --- | --- | --- |
| Actinobacteriota | RelAbundance | untreated | MH-treated | 5 | 5 | 25 | 0.00794 | 0.0265 |
| Crenarchaeota | RelAbundance | untreated | MH-treated | 5 | 5 | 25 | 0.00749 | 0.0265 |
| Firmicutes | RelAbundance | untreated | MH-treated | 5 | 5 | 0 | 0.00794 | 0.0265 |
| Chloroflexi | RelAbundance | untreated | MH-treated | 5 | 5 | 1 | 0.0159 | 0.0398 |
| Bacteroidota | RelAbundance | untreated | MH-treated | 5 | 5 | 4 | 0.0952 | 0.19 |
| Proteobacteria | RelAbundance | untreated | MH-treated | 5 | 5 | 5 | 0.151 | 0.252 |
| Acidobacteriota | RelAbundance | untreated | MH-treated | 5 | 5 | 7 | 0.31 | 0.443 |
| Verrucomicrobiota | RelAbundance | untreated | MH-treated | 5 | 5 | 8 | 0.421 | 0.526 |
| Planctomycetota | RelAbundance | untreated | MH-treated | 5 | 5 | 9 | 0.548 | 0.609 |
| Myxococcota | RelAbundance | untreated | MH-treated | 5 | 5 | 15 | 0.69 | 0.69 |

**Table S4**. Paired Wilcoxon signed-rank test of top 10 prokaryotic phyla between dysbiotic and native rhizospheres after planting

| Phylum | .y. | group1 | group2 | n1 | n2 | statistic | p | p.adj |
| --- | --- | --- | --- | --- | --- | --- | --- | --- |
| Acidobacteriota | RelAbundance | native | dysbiotic | 20 | 20 | 400 | 1.45E-11 | 3.62E-11 |
| Actinobacteriota | RelAbundance | native | dysbiotic | 20 | 20 | 400 | 1.45E-11 | 3.62E-11 |
| Firmicutes_D | RelAbundance | native | dysbiotic | 20 | 20 | 0 | 1.45E-11 | 3.62E-11 |
| Proteobacteria | RelAbundance | native | dysbiotic | 20 | 20 | 0 | 1.45E-11 | 3.62E-11 |
| Bacteroidota | RelAbundance | native | dysbiotic | 20 | 20 | 2 | 5.80E-11 | 1.16E-10 |
| Thermoproteota | RelAbundance | native | dysbiotic | 20 | 20 | 400 | 8.01E-09 | 1.33E-08 |
| p_un_d_Bacteria | RelAbundance | native | dysbiotic | 20 | 20 | 385 | 9.92E-09 | 1.42E-08 |
| Chloroflexota | RelAbundance | native | dysbiotic | 20 | 20 | 400 | 6.76E-08 | 8.45E-08 |
| Planctomycetota | RelAbundance | native | dysbiotic | 20 | 20 | 262 | 9.65E-02 | 1.07E-01 |
| Verrucomicrobiota | RelAbundance | native | dysbiotic | 20 | 20 | 241 | 2.77E-01 | 2.77E-01 |

**Table S5** Absolute abundance of prokaryotic communities in dysbiotic and native cucumber rhizospheres across microbial inoculations.

| Moist-heat  treatment |  | Microbial  inoculation | Mean Abundance  (x10^6^ cells/ g soil) | Standard Error Abundance |
| --- | --- | --- | --- | --- |
| Dysbiotic  Rhizosphere |  | bioagent | 34.84 | 6.39 |
|  |  | control | 32.27 | 3.11 |
|  |  | pathogen | 32.40 | 1.57 |
|  |  | Pat + bio | 31.38 | 5.71 |
| Native  Rhizosphere |  | bioagent | 132.69 | 19.55 |
|  |  | control | 151.44 | 25.83 |
|  |  | pathogen | 70.48 | 10.06 |
|  |  | Pat + bio | 135.95 | 12.80 |

**Table S6** Kruskal–Wallis test of relative abundances of the top 10 phyla among microbial treatments and control in dysbiotic rhizosphere

| Phylum | Variable | n | Statistic | df | p-value |
| --- | --- | --- | --- | --- | --- |
| Planctomycetota | RelAbundance | 20 | 17.9 | 3 | 0.000471 |
| Actinobacteriota | RelAbundance | 20 | 14.8 | 3 | 0.00196 |
| un_d__Bacteria | RelAbundance | 20 | 13.9 | 3 | 0.00302 |
| Bdellovibrionota | RelAbundance | 20 | 12.8 | 3 | 0.00508 |
| Firmicutes | RelAbundance | 20 | 12.7 | 3 | 0.00538 |
| Gemmatimonadota | RelAbundance | 20 | 12.6 | 3 | 0.00565 |
| Verrucomicrobiota | RelAbundance | 20 | 11.5 | 3 | 0.0093 |
| Proteobacteria | RelAbundance | 20 | 11 | 3 | 0.0119 |
| Myxococcota | RelAbundance | 20 | 9.86 | 3 | 0.0198 |
| Bacteroidota | RelAbundance | 20 | 4.78 | 3 | 0.188 |

**Table S7** Paired Wilcoxon signed-rank test of top 10 prokaryotic phyla among microbial treatments in dysbiotic rhizosphere

| Phylum | .y. | group1 | group2 | n1 | n2 | statistic | p | p.adj | p.adj.sig. | y.position |
| --- | --- | --- | --- | --- | --- | --- | --- | --- | --- | --- |
| Actinobacteriota | RelAbundance | bioagent | control | 5 | 5 | 0 | 0.008 | 0.02 | * | 14.9806 |
| Actinobacteriota | RelAbundance | bioagent | pathogen | 5 | 5 | 8 | 0.421 | 0.48 | ns | 14.9806 |
| Actinobacteriota | RelAbundance | bioagent | pat+bio | 5 | 5 | 22 | 0.056 | 0.09 | ns | 14.9806 |
| Actinobacteriota | RelAbundance | control | pathogen | 5 | 5 | 25 | 0.008 | 0.02 | * | 14.9806 |
| Actinobacteriota | RelAbundance | control | pat+bio | 5 | 5 | 25 | 0.008 | 0.02 | * | 14.9806 |
| Actinobacteriota | RelAbundance | pathogen | pat+bio | 5 | 5 | 24 | 0.016 | 0.04 | * | 14.9806 |
| Bacteroidota | RelAbundance | bioagent | control | 5 | 5 | 4 | 0.095 | 0.15 | ns | 13.53979 |
| Bacteroidota | RelAbundance | bioagent | pathogen | 5 | 5 | 10 | 0.69 | 0.70 | ns | 13.53979 |
| Bacteroidota | RelAbundance | bioagent | pat+bio | 5 | 5 | 7 | 0.31 | 0.38 | ns | 13.53979 |
| Bacteroidota | RelAbundance | control | pathogen | 5 | 5 | 20 | 0.151 | 0.21 | ns | 13.53979 |
| Bacteroidota | RelAbundance | control | pat+bio | 5 | 5 | 19 | 0.222 | 0.30 | ns | 13.53979 |
| Bacteroidota | RelAbundance | pathogen | pat+bio | 5 | 5 | 10 | 0.69 | 0.70 | ns | 13.53979 |
| Bdellovibrionota | RelAbundance | bioagent | control | 5 | 5 | 22 | 0.056 | 0.09 | ns | 2.823124 |
| Bdellovibrionota | RelAbundance | bioagent | pathogen | 5 | 5 | 24 | 0.016 | 0.04 | * | 2.823124 |
| Bdellovibrionota | RelAbundance | bioagent | pat+bio | 5 | 5 | 8 | 0.421 | 0.48 | ns | 2.823124 |
| Bdellovibrionota | RelAbundance | control | pathogen | 5 | 5 | 16 | 0.548 | 0.61 | ns | 2.823124 |
| Bdellovibrionota | RelAbundance | control | pat+bio | 5 | 5 | 0 | 0.008 | 0.02 | * | 2.823124 |
| Bdellovibrionota | RelAbundance | pathogen | pat+bio | 5 | 5 | 0 | 0.008 | 0.02 | * | 2.823124 |
| Firmicutes | RelAbundance | bioagent | control | 5 | 5 | 23 | 0.032 | 0.06 | ns | 37.10228 |
| Firmicutes | RelAbundance | bioagent | pathogen | 5 | 5 | 2 | 0.032 | 0.06 | ns | 37.10228 |
| Firmicutes | RelAbundance | bioagent | pat+bio | 5 | 5 | 4 | 0.095 | 0.15 | ns | 37.10228 |
| Firmicutes | RelAbundance | control | pathogen | 5 | 5 | 0 | 0.008 | 0.02 | * | 37.10228 |
| Firmicutes | RelAbundance | control | pat+bio | 5 | 5 | 1 | 0.016 | 0.04 | * | 37.10228 |
| Firmicutes | RelAbundance | pathogen | pat+bio | 5 | 5 | 7 | 0.31 | 0.38 | ns | 37.10228 |
| Gemmatimonadota | RelAbundance | bioagent | control | 5 | 5 | 23 | 0.032 | 0.06 | ns | 1.152283 |
| Gemmatimonadota | RelAbundance | bioagent | pathogen | 5 | 5 | 25 | 0.008 | 0.02 | * | 1.152283 |
| Gemmatimonadota | RelAbundance | bioagent | pat+bio | 5 | 5 | 6 | 0.222 | 0.30 | ns | 1.152283 |
| Gemmatimonadota | RelAbundance | control | pathogen | 5 | 5 | 11 | 0.841 | 0.84 | ns | 1.152283 |
| Gemmatimonadota | RelAbundance | control | pat+bio | 5 | 5 | 2 | 0.032 | 0.06 | ns | 1.152283 |
| Gemmatimonadota | RelAbundance | pathogen | pat+bio | 5 | 5 | 0 | 0.008 | 0.02 | * | 1.152283 |
| Myxococcota | RelAbundance | bioagent | control | 5 | 5 | 25 | 0.008 | 0.02 | * | 0.774535 |
| Myxococcota | RelAbundance | bioagent | pathogen | 5 | 5 | 25 | 0.008 | 0.02 | * | 0.774535 |
| Myxococcota | RelAbundance | bioagent | pat+bio | 5 | 5 | 15 | 0.69 | 0.70 | ns | 0.774535 |
| Myxococcota | RelAbundance | control | pathogen | 5 | 5 | 15 | 0.69 | 0.70 | ns | 0.774535 |
| Myxococcota | RelAbundance | control | pat+bio | 5 | 5 | 5 | 0.151 | 0.21 | ns | 0.774535 |
| Myxococcota | RelAbundance | pathogen | pat+bio | 5 | 5 | 5 | 0.151 | 0.21 | ns | 0.774535 |
| Planctomycetota | RelAbundance | bioagent | control | 5 | 5 | 25 | 0.008 | 0.02 | * | 5.713598 |
| Planctomycetota | RelAbundance | bioagent | pathogen | 5 | 5 | 25 | 0.008 | 0.02 | * | 5.713598 |
| Planctomycetota | RelAbundance | bioagent | pat+bio | 5 | 5 | 0 | 0.008 | 0.02 | * | 5.713598 |
| Planctomycetota | RelAbundance | control | pathogen | 5 | 5 | 25 | 0.008 | 0.02 | * | 5.713598 |
| Planctomycetota | RelAbundance | control | pat+bio | 5 | 5 | 0 | 0.008 | 0.02 | * | 5.713598 |
| Planctomycetota | RelAbundance | pathogen | pat+bio | 5 | 5 | 0 | 0.008 | 0.02 | * | 5.713598 |
| Proteobacteria | RelAbundance | bioagent | control | 5 | 5 | 8 | 0.421 | 0.48 | ns | 54.83372 |
| Proteobacteria | RelAbundance | bioagent | pathogen | 5 | 5 | 17 | 0.421 | 0.48 | ns | 54.83372 |
| Proteobacteria | RelAbundance | bioagent | pat+bio | 5 | 5 | 22 | 0.056 | 0.09 | ns | 54.83372 |
| Proteobacteria | RelAbundance | control | pathogen | 5 | 5 | 25 | 0.008 | 0.02 | * | 54.83372 |
| Proteobacteria | RelAbundance | control | pat+bio | 5 | 5 | 25 | 0.008 | 0.02 | * | 54.83372 |
| Proteobacteria | RelAbundance | pathogen | pat+bio | 5 | 5 | 20 | 0.151 | 0.21 | ns | 54.83372 |
| Verrucomicrobiota | RelAbundance | bioagent | control | 5 | 5 | 25 | 0.008 | 0.02 | * | 8.934821 |
| Verrucomicrobiota | RelAbundance | bioagent | pathogen | 5 | 5 | 25 | 0.008 | 0.02 | * | 8.934821 |
| Verrucomicrobiota | RelAbundance | bioagent | pat+bio | 5 | 5 | 18 | 0.31 | 0.38 | ns | 8.934821 |
| Verrucomicrobiota | RelAbundance | control | pathogen | 5 | 5 | 25 | 0.008 | 0.02 | * | 8.934821 |
| Verrucomicrobiota | RelAbundance | control | pat+bio | 5 | 5 | 10 | 0.69 | 0.70 | ns | 8.934821 |
| Verrucomicrobiota | RelAbundance | pathogen | pat+bio | 5 | 5 | 5 | 0.151 | 0.21 | ns | 8.934821 |
| un_d__Bacteria | RelAbundance | bioagent | control | 5 | 5 | 3 | 0.056 | 0.09 | ns | 4.008791 |
| un_d__Bacteria | RelAbundance | bioagent | pathogen | 5 | 5 | 0 | 0.008 | 0.02 | * | 4.008791 |
| un_d__Bacteria | RelAbundance | bioagent | pat+bio | 5 | 5 | 18 | 0.31 | 0.38 | ns | 4.008791 |
| un_d__Bacteria | RelAbundance | control | pathogen | 5 | 5 | 2 | 0.032 | 0.06 | ns | 4.008791 |
| un_d__Bacteria | RelAbundance | control | pat+bio | 5 | 5 | 23 | 0.032 | 0.06 | ns | 4.008791 |
| un_d__Bacteria | RelAbundance | pathogen | pat+bio | 5 | 5 | 25 | 0.008 | 0.02 | * | 4.008791 |

**Table S8** Kruskal–Wallis test of relative abundances of the top 10 phyla among microbial treatments and control in native rhizosphere

| Phylum | Variable | n | Mean | df | p-value |
| --- | --- | --- | --- | --- | --- |
| Chloroflexi | RelAbundance | 20 | 15.1 | 3 | 0.00176 |
| Acidobacteriota | RelAbundance | 20 | 14.8 | 3 | 0.00202 |
| Proteobacteria | RelAbundance | 20 | 12.8 | 3 | 0.00516 |
| Actinobacteriota | RelAbundance | 20 | 11.8 | 3 | 0.00819 |
| Crenarchaeota | RelAbundance | 20 | 10.6 | 3 | 0.0142 |
| Bacteroidota | RelAbundance | 20 | 10.3 | 3 | 0.0159 |
| Verrucomicrobiota | RelAbundance | 20 | 7.3 | 3 | 0.063 |
| Firmicutes | RelAbundance | 20 | 4.85 | 3 | 0.183 |
| Myxococcota | RelAbundance | 20 | 4.51 | 3 | 0.212 |
| Planctomycetota | RelAbundance | 20 | 3.53 | 3 | 0.317 |

**Table S9** Paired Wilcoxon signed-rank test of top 10 prokaryotic phyla among microbial treatments in native rhizosphere

| Phylum | .y. | group1 | group2 | n1 | n2 | statistic | p | p.adj | p.adj.sig. | y.position |
| --- | --- | --- | --- | --- | --- | --- | --- | --- | --- | --- |
| Acidobacteriota | RelAbundance | bioagent | control | 5 | 5 | 2 | 0.032 | 0.09 | ns | 11.63965 |
| Acidobacteriota | R``elAbundance | bioagent | pathogen | 5 | 5 | 0 | 0.008 | 0.04 | * | 11.63965 |
| Acidobacteriota | RelAbundance | bioagent | pat+bio | 5 | 5 | 22 | 0.056 | 0.14 | ns | 11.63965 |
| Acidobacteriota | RelAbundance | control | pathogen | 5 | 5 | 8 | 0.421 | 0.55 | ns | 11.63965 |
| Acidobacteriota | RelAbundance | control | pat+bio | 5 | 5 | 25 | 0.008 | 0.04 | * | 11.63965 |
| Acidobacteriota | RelAbundance | pathogen | pat+bio | 5 | 5 | 25 | 0.008 | 0.04 | * | 11.63965 |
| Actinobacteriota | RelAbundance | bioagent | control | 5 | 5 | 5 | 0.151 | 0.27 | ns | 19.79883 |
| Actinobacteriota | RelAbundance | bioagent | pathogen | 5 | 5 | 7 | 0.31 | 0.45 | ns | 19.79883 |
| Actinobacteriota | RelAbundance | bioagent | pat+bio | 5 | 5 | 24 | 0.016 | 0.05 | ns | 19.79883 |
| Actinobacteriota | RelAbundance | control | pathogen | 5 | 5 | 10 | 0.69 | 0.75 | ns | 19.79883 |
| Actinobacteriota | RelAbundance | control | pat+bio | 5 | 5 | 25 | 0.008 | 0.04 | * | 19.79883 |
| Actinobacteriota | RelAbundance | pathogen | pat+bio | 5 | 5 | 25 | 0.008 | 0.04 | * | 19.79883 |
| Bacteroidota | RelAbundance | bioagent | control | 5 | 5 | 9 | 0.548 | 0.63 | ns | 12.32916 |
| Bacteroidota | RelAbundance | bioagent | pathogen | 5 | 5 | 6 | 0.222 | 0.37 | ns | 12.32916 |
| Bacteroidota | RelAbundance | bioagent | pat+bio | 5 | 5 | 1 | 0.016 | 0.05 | ns | 12.32916 |
| Bacteroidota | RelAbundance | control | pathogen | 5 | 5 | 8 | 0.421 | 0.55 | ns | 12.32916 |
| Bacteroidota | RelAbundance | control | pat+bio | 5 | 5 | 1 | 0.016 | 0.05 | ns | 12.32916 |
| Bacteroidota | RelAbundance | pathogen | pat+bio | 5 | 5 | 1 | 0.016 | 0.05 | ns | 12.32916 |
| Chloroflexi | RelAbundance | bioagent | control | 5 | 5 | 0 | 0.008 | 0.04 | * | 7.479492 |
| Chloroflexi | RelAbundance | bioagent | pathogen | 5 | 5 | 0 | 0.008 | 0.04 | * | 7.479492 |
| Chloroflexi | RelAbundance | bioagent | pat+bio | 5 | 5 | 20 | 0.151 | 0.27 | ns | 7.479492 |
| Chloroflexi | RelAbundance | control | pathogen | 5 | 5 | 9 | 0.548 | 0.63 | ns | 7.479492 |
| Chloroflexi | RelAbundance | control | pat+bio | 5 | 5 | 25 | 0.008 | 0.04 | * | 7.479492 |
| Chloroflexi | RelAbundance | pathogen | pat+bio | 5 | 5 | 25 | 0.008 | 0.04 | * | 7.479492 |
| Crenarchaeota | RelAbundance | bioagent | control | 5 | 5 | 16 | 0.548 | 0.63 | ns | 8.682546 |
| Crenarchaeota | RelAbundance | bioagent | pathogen | 5 | 5 | 3 | 0.056 | 0.14 | ns | 8.682546 |
| Crenarchaeota | RelAbundance | bioagent | pat+bio | 5 | 5 | 19 | 0.222 | 0.37 | ns | 8.682546 |
| Crenarchaeota | RelAbundance | control | pathogen | 5 | 5 | 0 | 0.008 | 0.04 | * | 8.682546 |
| Crenarchaeota | RelAbundance | control | pat+bio | 5 | 5 | 16 | 0.548 | 0.63 | ns | 8.682546 |
| Crenarchaeota | RelAbundance | pathogen | pat+bio | 5 | 5 | 25 | 0.008 | 0.04 | * | 8.682546 |
| Firmicutes | RelAbundance | bioagent | control | 5 | 5 | 7 | 0.31 | 0.45 | ns | 12.45153 |
| Firmicutes | RelAbundance | bioagent | pathogen | 5 | 5 | 13 | 1 | 1.00 | ns | 12.45153 |
| Firmicutes | RelAbundance | bioagent | pat+bio | 5 | 5 | 14 | 0.841 | 0.90 | ns | 12.45153 |
| Firmicutes | RelAbundance | control | pathogen | 5 | 5 | 21 | 0.095 | 0.20 | ns | 12.45153 |
| Firmicutes | RelAbundance | control | pat+bio | 5 | 5 | 22 | 0.056 | 0.14 | ns | 12.45153 |
| Firmicutes | RelAbundance | pathogen | pat+bio | 5 | 5 | 18 | 0.31 | 0.45 | ns | 12.45153 |
| Myxococcota | RelAbundance | bioagent | control | 5 | 5 | 4 | 0.095 | 0.20 | ns | 3.476787 |
| Myxococcota | RelAbundance | bioagent | pathogen | 5 | 5 | 12 | 1 | 1.00 | ns | 3.476787 |
| Myxococcota | RelAbundance | bioagent | pat+bio | 5 | 5 | 15 | 0.69 | 0.75 | ns | 3.476787 |
| Myxococcota | RelAbundance | control | pathogen | 5 | 5 | 20 | 0.151 | 0.27 | ns | 3.476787 |
| Myxococcota | RelAbundance | control | pat+bio | 5 | 5 | 20 | 0.151 | 0.27 | ns | 3.476787 |
| Myxococcota | RelAbundance | pathogen | pat+bio | 5 | 5 | 16 | 0.548 | 0.63 | ns | 3.476787 |
| Planctomycetota | RelAbundance | bioagent | control | 5 | 5 | 17 | 0.421 | 0.55 | ns | 6.560921 |
| Planctomycetota | RelAbundance | bioagent | pathogen | 5 | 5 | 12 | 1 | 1 | ns | 6.560921 |
| Planctomycetota | RelAbundance | bioagent | pat+bio | 5 | 5 | 7 | 0.31 | 0.453659 | ns | 6.560921 |
| Planctomycetota | RelAbundance | control | pathogen | 5 | 5 | 7 | 0.31 | 0.453659 | ns | 6.560921 |
| Planctomycetota | RelAbundance | control | pat+bio | 5 | 5 | 5 | 0.151 | 0.274545 | ns | 6.560921 |
| Planctomycetota | RelAbundance | pathogen | pat+bio | 5 | 5 | 8 | 0.421 | 0.54913 | ns | 6.560921 |
| Proteobacteria | RelAbundance | bioagent | control | 5 | 5 | 23 | 0.032 | 0.091429 | ns | 40.27604 |
| Proteobacteria | RelAbundance | bioagent | pathogen | 5 | 5 | 25 | 0.008 | 0.036923 | * | 40.27604 |
| Proteobacteria | RelAbundance | bioagent | pat+bio | 5 | 5 | 10 | 0.69 | 0.752727 | ns | 40.27604 |
| Proteobacteria | RelAbundance | control | pathogen | 5 | 5 | 13 | 1 | 1 | ns | 40.27604 |
| Proteobacteria | RelAbundance | control | pat+bio | 5 | 5 | 1 | 0.016 | 0.053333 | ns | 40.27604 |
| Proteobacteria | RelAbundance | pathogen | pat+bio | 5 | 5 | 0 | 0.008 | 0.036923 | * | 40.27604 |
| Verrucomicrobiota | RelAbundance | bioagent | control | 5 | 5 | 21 | 0.095 | 0.203571 | ns | 5.577178 |
| Verrucomicrobiota | RelAbundance | bioagent | pathogen | 5 | 5 | 9 | 0.548 | 0.632308 | ns | 5.577178 |
| Verrucomicrobiota | RelAbundance | bioagent | pat+bio | 5 | 5 | 6 | 0.222 | 0.37 | ns | 5.577178 |
| Verrucomicrobiota | RelAbundance | control | pathogen | 5 | 5 | 4 | 0.095 | 0.203571 | ns | 5.577178 |
| Verrucomicrobiota | RelAbundance | control | pat+bio | 5 | 5 | 2 | 0.032 | 0.091429 | ns | 5.577178 |
| Verrucomicrobiota | RelAbundance | pathogen | pat+bio | 5 | 5 | 8 | 0.421 | 0.54913 | ns | 5.577178 |

**Table S10** Summary of RNA-seq read alignment to the reference genome

| **Metric** | **Count** | **Percentage** |
| --- | --- | --- |
| **Total reads** | **37,431,665** | **100%** |
| **Paired reads** | **34,386,784** | **91.87%** |
| - Concordantly aligned exactly 1 time | 27,727,118 | 80.63% |
| - Concordantly aligned >1 times | 1,611,836 | 4.69% |
| - Concordantly aligned 0 times | 5,047,830 | 14.68% |
| **Unpaired reads** | **3,044,881** | **8.13%** |
| - Aligned exactly 1 time | 2,302,873 | 75.63% |
| - Aligned >1 times | 205,961 | 6.76% |
| - Aligned 0 times | 536,047 | 17.60% |
| **Overall alignment rate (HISAT2 summary)** | — | 89.27% |

**Table S11:** Gene ontology (GO) terms and enrichment analysis indicating the enrichment ratios of differentially expressed genes

| GO_term | Term_description | Ontology | Gene_count | Background  count | p_value | enrichment_  ratio |
| --- | --- | --- | --- | --- | --- | --- |
| GO:0009800 | cinnamic acid biosynthetic process | BP | 7 | 15 | 4.20E-07 | 0.466667 |
| GO:0045548 | phenylalanine ammonia-lyase activity | MF | 7 | 15 | 4.20E-07 | 0.466667 |
| GO:0006559 | L-phenylalanine catabolic process | BP | 7 | 20 | 4.31E-06 | 0.35 |
| GO:0004535 | poly(A)-specific ribonuclease activity | MF | 3 | 10 | 0.004809 | 0.3 |
| GO:0043928 | exonucleolytic catabolism of deadenylated mRNA | BP | 3 | 13 | 0.010562 | 0.230769 |
| GO:0032440 | 2-alkenal reductase [NAD(P)+] activity | MF | 4 | 25 | 0.012133 | 0.16 |
| GO:0004014 | adenosylmethionine decarboxylase activity | MF | 2 | 5 | 0.012389 | 0.4 |
| GO:0006557 | NA | NA | 2 | 5 | 0.012389 | 0.4 |
| GO:0006597 | spermine biosynthetic process | BP | 2 | 5 | 0.012389 | 0.4 |
| GO:2000762 | regulation of phenylpropanoid metabolic process | BP | 2 | 5 | 0.012389 | 0.4 |
| GO:0030015 | CCR4-NOT core complex | CC | 3 | 15 | 0.015916 | 0.2 |
| GO:0005987 | sucrose catabolic process | BP | 2 | 6 | 0.018136 | 0.333333 |
| GO:0008295 | spermidine biosynthetic process | BP | 2 | 6 | 0.018136 | 0.333333 |
| GO:0005262 | calcium channel activity | MF | 2 | 7 | 0.024781 | 0.285714 |
| GO:0033926 | glycopeptide alpha-N-acetylgalactosaminidase activity | MF | 2 | 7 | 0.024781 | 0.285714 |
| GO:0034620 | cellular response to unfolded protein | BP | 2 | 7 | 0.024781 | 0.285714 |
| GO:0034647 | histone H3K4me/H3K4me2/H3K4me3 demethylase activity | MF | 2 | 7 | 0.024781 | 0.285714 |
| GO:0034721 | histone H3-K4 demethylation, trimethyl-H3-K4-specific | BP | 2 | 7 | 0.024781 | 0.285714 |
| GO:0044390 | ubiquitin-like protein conjugating enzyme binding | MF | 2 | 7 | 0.024781 | 0.285714 |
| GO:0004575 | sucrose alpha-glucosidase activity | MF | 2 | 8 | 0.032251 | 0.25 |
| GO:0042594 | response to starvation | BP | 2 | 8 | 0.032251 | 0.25 |
| GO:0006000 | fructose metabolic process | BP | 2 | 9 | 0.040477 | 0.222222 |
| GO:0009725 | response to hormone | BP | 2 | 9 | 0.040477 | 0.222222 |
| GO:0016868 | intramolecular transferase activity, phosphotransferases | MF | 2 | 9 | 0.040477 | 0.222222 |
| GO:0071586 | CAAX-box protein processing | BP | 2 | 9 | 0.040477 | 0.222222 |
| GO:0030176 | NA | NA | 4 | 36 | 0.041301 | 0.111111 |

**Table S12**. List of differentially expressed genes (DEGs) identified by DESeq2 with corresponding expression statistics

| ENSEMBL | baseMean | log2FoldChange | lfcSE | stat | pvalue | padj | description |
| --- | --- | --- | --- | --- | --- | --- | --- |
| CsaV3_3G002860 | 129.733652 | -3.218031513 | 0.39138 | -8.22227 | 2.00E-16 | 1.80E-12 | 14 kDa proline-rich protein DC2.15-like |
| CsaV3_4G032800 | 30.91055067 | 8.368094503 | 1.094166 | 7.647919 | 2.04E-14 | 9.19E-11 | Unknown protein |
| CsaV3_6G050470 | 22.88915992 | -7.983329454 | 1.08718 | -7.34315 | 2.09E-13 | 6.26E-10 | MLO-like protein |
| CsaV3_3G035020 | 90.55424254 | -1.652095913 | 0.331992 | -4.97632 | 6.48E-07 | 0.001165975 | short-chain dehydrogenase TIC 32, chloroplastic |
| CsaV3_2G030370 | 287.7103932 | -1.504306696 | 0.345053 | -4.35964 | 1.30E-05 | 0.016742043 | Eukaryotic aspartyl protease family protein |
| CsaV3_7G027600 | 134.3776775 | -1.321779697 | 0.312684 | -4.22721 | 2.37E-05 | 0.023650169 | Auxin-responsive protein |
| CsaV3_6G014230 | 235.7469284 | -1.297443093 | 0.3145 | -4.12541 | 3.70E-05 | 0.033291943 | heavy metal-associated isoprenylated plant protein 3-like |
| CsaV3_3G038750 | 39.14471725 | 8.713852145 | 2.14528 | 4.061872 | 4.87E-05 | 0.039811996 | metal tolerance protein C2 |
| CsaV3_1G038820 | 509.6149292 | -1.114279836 | 0.281045 | -3.96477 | 7.35E-05 | 0.050838043 | dormancy-associated protein homolog 3-like isoform X2 |
| CsaV3_7G033950 | 36.22409113 | 8.601555879 | 2.18152 | 3.942918 | 8.05E-05 | 0.051724449 | Unknown protein |
| CsaV3_3G044690 | 32.13214286 | 8.428933598 | 2.205449 | 3.821868 | 0.000132 | 0.066192816 | Cytochrome P450 |
| CsaV3_3G032730 | 145.162115 | 1.023723281 | 0.26908 | 3.804534 | 0.000142 | 0.067267064 | Chaperone protein dnaJ 8 |
| CsaV3_6G007150 | 25.28726865 | 8.083244577 | 2.18384 | 3.70139 | 0.000214 | 0.087678898 | dynamin-like protein |
| CsaV3_7G006370 | 534.8601088 | -1.240112308 | 0.339257 | -3.65538 | 0.000257 | 0.099341417 | Peroxidase |
| CsaV3_1G036900 | 275.0890149 | -1.605858152 | 0.44094 | -3.6419 | 0.000271 | 0.099341417 | phospholipase A1-Igamma1, chloroplastic |
| CsaV3_5G040440 | 22.45722943 | 7.912008428 | 2.182968 | 3.624428 | 0.00029 | 0.100202344 | Fanconi-associated nuclease |
| CsaV3_4G032810 | 23.26919441 | 7.962897338 | 2.208122 | 3.606185 | 0.000311 | 0.103531156 | Bidirectional sugar transporter SWEET |
| CsaV3_1G009400 | 15.21815813 | -7.409035532 | 2.130952 | -3.47687 | 0.000507 | 0.152125548 | Unknown protein |
| CsaV3_3G041280 | 22.86440058 | 7.937052042 | 2.300353 | 3.450363 | 0.00056 | 0.155060303 | NAC domain-containing protein |
| CsaV3_7G028200 | 18.18409233 | 7.607223557 | 2.209555 | 3.442876 | 0.000576 | 0.155060303 | General transcription and DNA repair factor IIH helicase subunit XPD |
| CsaV3_6G018860 | 447.2019973 | -1.218359559 | 0.354381 | -3.43799 | 0.000586 | 0.155060303 | Trichome birefringence-like family |
| CsaV3_3G042200 | 121.697851 | -1.166396421 | 0.342832 | -3.40224 | 0.000668 | 0.169129342 | Potassium transporter |
| CsaV3_3G041480 | 13.79062762 | -7.267316942 | 2.144631 | -3.38861 | 0.000702 | 0.170797734 | Gibberellin regulated protein |
| CsaV3_2G003620 | 313.7134429 | -1.279937087 | 0.380304 | -3.36556 | 0.000764 | 0.180837789 | Serine-rich protein-like protein |
| CsaV3_3G042040 | 12.56816223 | -7.13387938 | 2.125026 | -3.35708 | 0.000788 | 0.181697395 | Peroxidase |
| CsaV3_4G026530 | 152.2420331 | 1.035838145 | 0.312947 | 3.309945 | 0.000933 | 0.199870052 | abscisic acid receptor PYL4 |
| CsaV3_3G009190 | 144.4292141 | -1.390673482 | 0.422693 | -3.29003 | 0.001002 | 0.203664791 | BON1-associated protein 2-like |
| CsaV3_7G026270 | 11.54908904 | -7.012203593 | 2.134425 | -3.28529 | 0.001019 | 0.203664791 | ribonuclease 1-like |
| CsaV3_7G026490 | 13.78606024 | 7.208448727 | 2.234162 | 3.226467 | 0.001253 | 0.234155238 | Protein of unknown function (DUF506) |
| CsaV3_5G024290 | 13.35121509 | 7.16267288 | 2.22493 | 3.21928 | 0.001285 | 0.234155238 | rRNA N-glycosidase |
| CsaV3_7G028160 | 14.55145692 | 7.28481321 | 2.275746 | 3.201066 | 0.001369 | 0.24151673 | Pentatricopeptide repeat-containing protein |
| CsaV3_3G036090 | 12.15748064 | 7.028002387 | 2.234284 | 3.145527 | 0.001658 | 0.268195435 | Protein NUCLEAR FUSION DEFECTIVE 4-like |
| CsaV3_6G042480 | 12.93899039 | 7.115645114 | 2.267312 | 3.138362 | 0.001699 | 0.268195435 | Calcium uniporter protein |
| CsaV3_3G039370 | 150.4812597 | -1.320761461 | 0.42105 | -3.13683 | 0.001708 | 0.268195435 | Unknown protein |
| CsaV3_3G036480 | 12.97751328 | 7.11998929 | 2.274762 | 3.129993 | 0.001748 | 0.268195435 | PLATZ transcription factor family protein |
| CsaV3_2G012780 | 237.4847494 | -1.030771544 | 0.330551 | -3.11834 | 0.001819 | 0.272685187 | Myb-related transcription factor |
| CsaV3_4G017680 | 12.18575614 | -7.081313804 | 2.289285 | -3.09324 | 0.00198 | 0.278288546 | Unknown protein |
| CsaV3_4G033520 | 11.66675959 | 6.966514951 | 2.262114 | 3.079648 | 0.002072 | 0.286827955 | PRA1 family protein |
| CsaV3_5G026480 | 8.801953427 | -6.621816087 | 2.151697 | -3.07748 | 0.002088 | NA | RING-type domain-containing protein |
| CsaV3_7G033720 | 9.135479375 | -6.675175444 | 2.18307 | -3.0577 | 0.00223 | NA | 7-dehydrocholesterol reductase |
| CsaV3_7G006570 | 11.4357302 | 6.93750299 | 2.269346 | 3.057049 | 0.002235 | 0.3046751 | metacaspase-1 |
| CsaV3_1G007570 | 36.51768189 | -1.231996668 | 0.405921 | -3.03507 | 0.002405 | 0.312242578 | kunitz trypsin inhibitor 2 |
| CsaV3_1G040390 | 8.301297614 | -6.53759789 | 2.160825 | -3.02551 | 0.002482 | NA | GATA transcription factor 21 |
| CsaV3_3G039670 | 11.29421333 | -6.972811563 | 2.323129 | -3.00147 | 0.002687 | 0.335695555 | MYB transcription factor |
| CsaV3_2G011320 | 212.2813853 | 1.528691444 | 0.512471 | 2.98298 | 0.002855 | 0.337890812 | Auxin-regulated gene involved in organ size |
| CsaV3_7G001010 | 103.7967304 | 2.21916972 | 0.748088 | 2.966456 | 0.003013 | 0.351957382 | heavy metal-associated isoprenylated plant protein 26-like |
| CsaV3_7G005720 | 85.9988473 | -1.698883658 | 0.578214 | -2.93816 | 0.003302 | 0.375977582 | Peroxidase |
| CsaV3_1G039440 | 7.394385463 | -6.371353947 | 2.170324 | -2.93567 | 0.003328 | NA | vesicle-associated protein 2-2-like |
| CsaV3_3G049830 | 8.022212439 | -6.487713554 | 2.220756 | -2.9214 | 0.003485 | NA | Golgin subfamily A member 6-like protein 4, putative |
| CsaV3_5G004720 | 9.469191354 | 6.665384479 | 2.282794 | 2.919836 | 0.003502 | NA | Procollagen-proline 4-dioxygenase |
| CsaV3_2G030400 | 685.2813888 | -1.001807451 | 0.344895 | -2.90467 | 0.003676 | 0.4033235 | rho GTPase-activating protein REN1 |
| CsaV3_3G012760 | 284.4544863 | -1.243320702 | 0.428966 | -2.89842 | 0.003751 | 0.406503026 | Protein of unknown function (DUF506) |
| CsaV3_6G049230 | 8.407197745 | 2.007386556 | 0.692951 | 2.896867 | 0.003769 | NA | Unknown protein |
| CsaV3_3G009800 | 7.911239813 | 6.408612371 | 2.250631 | 2.847473 | 0.004407 | NA | DNA repair protein RAD51 homolog 3 isoform X3 |
| CsaV3_6G023940 | 8.883053403 | 6.572764307 | 2.314935 | 2.839287 | 0.004521 | NA | UDP-glucuronate decarboxylase |
| CsaV3_6G042590 | 96.87486937 | -1.011244812 | 0.358058 | -2.82425 | 0.004739 | 0.473707881 | protein DA1-related 1-like |
| CsaV3_3G025030 | 19.1836296 | 1.448865028 | 0.530105 | 2.733168 | 0.006273 | 0.539745634 | Mucin-5AC like |
| CsaV3_3G039100 | 162.3991485 | -1.348366613 | 0.49687 | -2.71372 | 0.006653 | 0.539745634 | Basic helix-loop-helix transcription factor |
| CsaV3_5G004900 | 551.5218411 | -1.093779312 | 0.418086 | -2.61616 | 0.008892 | 0.650378686 | ethylene-responsive transcription factor ERF105 |
| CsaV3_2G002300 | 117.0779798 | -1.29730898 | 0.501104 | -2.5889 | 0.009628 | 0.665381947 | Arabinogalactan protein 22, putative |
| CsaV3_5G001540 | 122.1673017 | -1.120082721 | 0.435412 | -2.57247 | 0.010098 | 0.67790185 | Unknown protein |
| CsaV3_7G002960 | 564.0713131 | -1.249957065 | 0.491418 | -2.54357 | 0.010972 | 0.695371924 | Glycine rich protein |
| CsaV3_6G044910 | 32.77950174 | 8.457455061 | 3.325187 | 2.543452 | 0.010976 | 0.695371924 | UDP-glycosyltransferase 91A1-like |
| CsaV3_3G044870 | 115.2211148 | -1.062907351 | 0.420463 | -2.52794 | 0.011473 | 0.712165854 | Hexosyltransferase |
| CsaV3_6G003540 | 91.15281806 | -1.383684565 | 0.547916 | -2.52536 | 0.011558 | 0.712165854 | zinc finger protein CONSTANS-LIKE 6 |
| CsaV3_2G007750 | 256.4521752 | -1.395688947 | 0.554702 | -2.51611 | 0.011866 | 0.72616421 | S-adenosylmethionine decarboxylase proenzyme |
| CsaV3_3G026280 | 5.955777737 | 5.995467867 | 2.389586 | 2.508998 | 0.012107 | NA | Auxin-responsive protein |
| CsaV3_3G044220 | 102.6950407 | 1.726141275 | 0.688605 | 2.506722 | 0.012186 | 0.731828634 | B box-type domain-containing protein |
| CsaV3_5G034410 | 230.9273129 | 1.040730795 | 0.420208 | 2.476703 | 0.01326 | 0.75499284 | UV excision repair protein |
| CsaV3_5G030700 | 5.453573865 | 5.868389561 | 2.373341 | 2.472629 | 0.013412 | NA | Cold-inducible RNA binding protein |
| CsaV3_1G009120 | 691.9938068 | 1.083754766 | 0.438407 | 2.472029 | 0.013435 | 0.760125355 | BTB/POZ and TAZ domain-containing protein 1 |
| CsaV3_4G033300 | 26.9122313 | 8.172759296 | 3.384788 | 2.414556 | 0.015754 | 0.821482331 | Glutamate receptor |
| CsaV3_5G034260 | 23.74215778 | -8.04778827 | 3.343439 | -2.40704 | 0.016082 | 0.825754867 | alpha-xylosidase 1-like |
| CsaV3_5G001690 | 216.7375509 | -1.025520037 | 0.42724 | -2.40034 | 0.01638 | 0.827831883 | Metal-nicotianamine transporter YSL1-like |
| CsaV3_6G031910 | 192.4875543 | 1.266464375 | 0.541802 | 2.337505 | 0.019413 | 0.913979212 | Damaged dna-binding 2, putative isoform 1 |
| CsaV3_3G032850 | 304.1325501 | -1.094511595 | 0.468601 | -2.3357 | 0.019507 | 0.913979212 | cinnamoyl coa reductase |
| CsaV3_1G041830 | 3.588117984 | -5.333707795 | 2.293112 | -2.32597 | 0.02002 | NA | Peptidyl-tRNA hydrolase family protein |
| CsaV3_7G033120 | 21.37721283 | 7.840552035 | 3.385155 | 2.316158 | 0.02055 | 0.922826078 | Sulfate transporter, putative |
| CsaV3_5G037960 | 19.5762427 | -7.76958172 | 3.355774 | -2.31529 | 0.020597 | 0.922826078 | ABC transporter B family member 19 |
| CsaV3_7G027960 | 71.91641766 | -1.143613935 | 0.494669 | -2.31188 | 0.020784 | 0.922826078 | FLZ-type domain-containing protein |
| CsaV3_7G032470 | 4.706263535 | -5.700435189 | 2.493271 | -2.28633 | 0.022235 | NA | Cytochrome P450 family protein |
| CsaV3_3G032830 | 270.5173527 | -1.026574692 | 0.455166 | -2.25538 | 0.024109 | 0.989131573 | cinnamoyl coa reductase |
| CsaV3_3G033870 | 3.993145798 | 5.41988231 | 2.405838 | 2.252804 | 0.024271 | NA | Mitochondrial inner membrane protease subunit 2 |
| CsaV3_6G029740 | 23.10482059 | -1.09415364 | 0.489582 | -2.23488 | 0.025426 | 0.992455708 | Integrase |
| CsaV3_3G048220 | 16.30147843 | 7.449584627 | 3.38571 | 2.200302 | 0.027785 | 0.999812323 | Phosphoglycerate mutase family protein |
| CsaV3_2G002520 | 15.21314741 | -7.406025422 | 3.386129 | -2.18717 | 0.02873 | 0.999812323 | ABC transporter G family member 32 |
| CsaV3_5G031900 | 14.99234114 | 7.329815288 | 3.385913 | 2.164797 | 0.030403 | 0.999812323 | IAA-amino acid hydrolase ILR1-like 4 |
| CsaV3_4G032530 | 14.9908199 | 7.328086427 | 3.385916 | 2.164285 | 0.030442 | 0.999812323 | Glycosyltransferase |
| CsaV3_2G007760 | 25.11373913 | -1.475024804 | 0.682445 | -2.16138 | 0.030666 | 0.999812323 | S-adenosylmethionine decarboxylase proenzyme-like |
| CsaV3_4G006480 | 659.3312531 | -1.210705136 | 0.561112 | -2.15769 | 0.030952 | 0.999812323 | WRKY domain-containing protein |
| CsaV3_6G047150 | 348.7651445 | 1.475619827 | 0.691167 | 2.13497 | 0.032763 | 0.999812323 | DnaJ-like protein |
| CsaV3_2G013760 | 3.610418465 | 5.271976607 | 2.474683 | 2.130364 | 0.033142 | NA | Unknown protein |
| CsaV3_6G015290 | 3.600213793 | 5.267944596 | 2.484369 | 2.120435 | 0.033969 | NA | Unknown protein |
| CsaV3_6G001850 | 13.50145345 | 7.177387583 | 3.386196 | 2.119602 | 0.03404 | 0.999812323 | NF-X1-type zinc finger protein NFXL2 |
| CsaV3_6G002710 | 106.3775845 | -1.067091029 | 0.503992 | -2.11728 | 0.034236 | 0.999812323 | Protein kinase domain-containing protein |
| CsaV3_1G028400 | 13.33891849 | 7.161233087 | 3.386228 | 2.114811 | 0.034446 | 0.999812323 | Flavin-containing monooxygenase |
| CsaV3_5G034940 | 13.14317146 | 7.139144652 | 3.386272 | 2.108261 | 0.035008 | 0.999812323 | S-adenosyl-L-methionine-dependent methyltransferases superfamily protein |
| CsaV3_3G009330 | 12.93943775 | 7.117261348 | 3.386316 | 2.101771 | 0.035573 | 0.999812323 | UDP-galactose/UDP-glucose transporter 2-like |
| CsaV3_1G006570 | 12.71026886 | 7.090147969 | 3.386372 | 2.09373 | 0.036284 | 0.999812323 | Unknown protein |
| CsaV3_2G004860 | 10.39445741 | -2.427357544 | 1.160931 | -2.09087 | 0.03654 | 0.999812323 | TIR-NBS-LRR disease resistance protein |
| CsaV3_3G047110 | 11.79601935 | -7.039507465 | 3.386925 | -2.07844 | 0.037669 | 0.999812323 | RING-type domain-containing protein |
| CsaV3_7G007290 | 3.100955302 | 5.058563196 | 2.43766 | 2.075172 | 0.037971 | NA | Unknown protein |
| CsaV3_3G044520 | 62.41524897 | -1.20122919 | 0.579597 | -2.07252 | 0.038217 | 0.999812323 | NAC domain protein |
| CsaV3_4G037030 | 11.47530049 | 6.942439732 | 3.386696 | 2.049915 | 0.040373 | 0.999812323 | Alpha-amylase |
| CsaV3_5G038980 | 10.85152473 | -6.919215415 | 3.387234 | -2.04273 | 0.041079 | 0.999812323 | protein trichome birefringence-like 19 |
| CsaV3_7G029990 | 11.23014093 | 6.912331496 | 3.386766 | 2.040983 | 0.041253 | 0.999812323 | GRAS domain-containing protein |
| CsaV3_3G014750 | 23.91154331 | -3.473869709 | 1.708958 | -2.03274 | 0.042079 | 0.999812323 | phylloplanin-like |
| CsaV3_4G004380 | 349.5451376 | 1.031824642 | 0.510052 | 2.022979 | 0.043075 | 0.999812323 | Glutamate dehydrogenase |
| CsaV3_4G001050 | 110.7021938 | -1.044142383 | 0.51643 | -2.02185 | 0.043192 | 0.999812323 | Hexosyltransferase |
| CsaV3_3G046430 | 1026.875654 | 1.210218703 | 0.601237 | 2.012882 | 0.044127 | 0.999812323 | EIN3-binding F-box protein 1 |
| CsaV3_1G002050 | 10.28241949 | 6.784921146 | 3.387079 | 2.003178 | 0.045158 | 0.999812323 | protein NUCLEAR FUSION DEFECTIVE 4 |
| CsaV3_4G008120 | 166.5936613 | 1.260734721 | 0.631895 | 1.995166 | 0.046025 | 0.999812323 | DUF641 domain-containing protein |
| CsaV3_7G007620 | 9.740072434 | 6.706265711 | 3.387287 | 1.979834 | 0.047722 | 0.999812323 | pathogenesis-related protein 1-like |
| CsaV3_6G045530 | 9.67297201 | 6.696507975 | 3.387313 | 1.976938 | 0.048049 | 0.999812323 | protein SCO1 homolog 2, mitochondrial |
| CsaV3_UNG071590 | 2.831021941 | 4.924573215 | 2.49141 | 1.976621 | 0.048084 | NA | Malic enzyme |
| CsaV3_1G001070 | 9.090193045 | -6.663989413 | 3.38798 | -1.96695 | 0.049189 | NA | Protein kinase domain-containing protein |
| CsaV3_6G018730 | 15.99821802 | -1.03864065 | 0.529396 | -1.96194 | 0.04977 | 0.999812323 | LINE-1 retrotransposable element ORF2 protein |
| CsaV3_3G015330 | 8.946786183 | -6.641041269 | 3.388054 | -1.96013 | 0.04998 | NA | transcription factor MYB108 |
